# Targeting Small GTPases and Their Prenylation in Diabetes Mellitus

**DOI:** 10.1021/acs.jmedchem.1c00410

**Published:** 2021-07-08

**Authors:** Edyta Gendaszewska-Darmach, Malgorzata A. Garstka, Katarzyna M. Błażewska

**Affiliations:** † Institute of Molecular and Industrial Biotechnology, Faculty of Biotechnology and Food Sciences, Lodz University of Technology, Stefanowskiego Street 4/10, 90-924 Łódź, Poland; ‡ Core Research Laboratory, Department of Endocrinology, Department of Tumor and Immunology, Precision Medical Institute, Western China Science and Technology Innovation Port, School of Medicine, the Second Affiliated Hospital of Xi’an Jiaotong University, DaMingGong, Jian Qiang Road, Wei Yang district, Xi’an 710016, China; § Institute of Organic Chemistry, Faculty of Chemistry, Lodz University of Technology, Żeromskiego Street 116, 90-924 Łódź, Poland

## Abstract

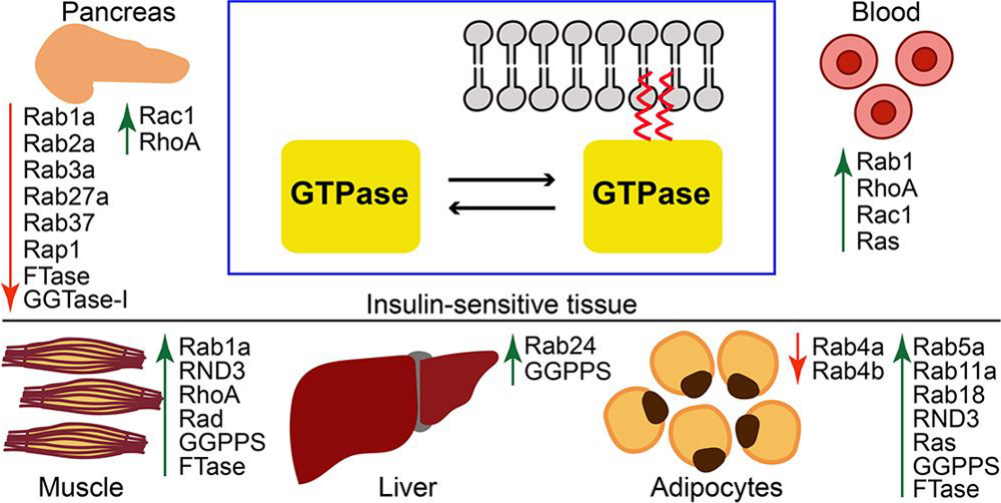

A fundamental role of pancreatic β-cells to maintain proper blood glucose level is controlled by the Ras superfamily of small GTPases that undergo post-translational modifications, including prenylation. This covalent attachment with either a farnesyl or a geranylgeranyl group controls their localization, activity, and protein–protein interactions. Small GTPases are critical in maintaining glucose homeostasis acting in the pancreas and metabolically active tissues such as skeletal muscles, liver, or adipocytes. Hyperglycemia-induced upregulation of small GTPases suggests that inhibition of these pathways deserves to be considered as a potential therapeutic approach in treating T2D. This Perspective presents how inhibition of various points in the mevalonate pathway might affect protein prenylation and functioning of diabetes-affected tissues and contribute to chronic inflammation involved in diabetes mellitus (T2D) development. We also demonstrate the currently available molecular tools to decipher the mechanisms linking the mevalonate pathway’s enzymes and GTPases with diabetes.

## 1 Introduction

The incidence of diabetes has increased tremendously over the last 50 years, affecting approximately 463 million adults. By 2045, there will be 700 million patients with diabetes.^1^ This epidemic is predominantly caused by a rise in the prevalence of type 2 diabetes (T2D), a complex disorder that is characterized by pancreatic β-cell failure with up to 50% cell loss at diagnosis coupled with impaired insulin sensitivity of target tissues, termed insulin resistance (IR). Initially, insulin resistance causes β-cells to secrete more insulin as a way to compensate for the deficiency. Increased metabolic activity of β-cells leads to the formation of reactive oxygen species (ROS) and induction of endoplasmic reticulum (ER) stress that promote inflammation. Initially, a low-grade local inflammation exerts favorable effects, inducing β-cell proliferation and insulin secretion. However, prolonged secretion of inflammatory mediators by β-cells results in proliferation of resident macrophages and recruitment of immune cells from the circulation. Immune cells further contribute to the inflammation that impairs β-cells function and leads to exhaustion.^2^

Enhanced insulin production results in hyperinsulinemia that promotes de novo lipogenesis, hyperlipidemia, and adipose tissue expansion. Expanded adipose tissue supports local and systemic inflammation by enhancing pro-inflammatory mediators secretion, including cytokines, chemokines, and adipokines. Both increased systemic fat and inflammation contribute to the development of IR in the liver and skeletal muscles. Insulin resistance can be observed decades before T2D onset and, together with low-grade chronic inflammation, represents one of the earliest pathogenic events in diabetes-related complications, including cardiovascular disease, diabetic retinopathy, and diabetic kidney disease (DKD) as well as nonalcoholic fatty liver disease (NAFLD). Moreover, insulin resistance, hyperinsulinemia, hyperglycemia, and chronic inflammation are the mechanisms of T2D-associated cancer occurrence and progression.^3^ Despite the large panel of treatment options for T2D, including insulin analogues, biguanides, meglitinides, sodium-glucose cotransporter-2 inhibitors, incretin-based therapies, dipeptidyl peptidase 4, α-glucosidase inhibitors, thiazolidinediones, and sulfonylureas, currently available therapies cause side effects and none of them have shown promise in halting the underlying causes of T2D, namely, insulin resistance.^4^

The factors associated with IR, T2D and related comorbidities are complex. However, altered activity and prenylation of small GTPases appears to constitute the link with the pathogenesis. Protein prenylation by isoprenoid groups is a crucial eukaryotic post-translational modification (PTM) of lipids predicted to affect hundreds of proteins in the human proteome.^5^ This ubiquitous covalent attachment of farnesyl or geranylgeranyl modulates localization and function of the plethora of signaling proteins. Most prenylated proteins belong to the Ras-related G proteins, particularly Ras, Rab, and Rho that control cell growth, differentiation, proliferation, biomolecule synthesis, and membrane trafficking.^6^ Of interest in this regard, hyperinsulinemia was shown to upregulate prenyltransferases,^7^ and selective inhibitors of prenylation markedly increased insulin sensitivity.^8,9^ Moreover, sustained inflammation-induced prenylation of Rho GTPase mediated inhibition of insulin-promoted glucose uptake, causing fasting hyperglycemia.^10^

The isoprenoids used for prenylation are produced by the mevalonate pathway, which is also responsible for cholesterol generation and can be blocked by statins, inhibitors of 3-hydroxymethyl-3-methylglutaryl coenzyme A (HMG-CoA) reductase. Moreover, statins hamper the production of downstream intermediates, such as FPP (farnesyl pyrophosphate) and GGPP (GRG, geranylgeranyl pyrophosphate, geranylgeranyl diphosphate). However, although statins were reported to improve insulin resistance and reduce systemic inflammation, some studies have shown that statins might have increased the incidence of diabetes.^11^ Farnesyl diphosphate synthase (FPPS) and geranylgeranyl diphosphate synthase (GGPPS), downstream of HMG-CoA reductase, catalyze the production of FPP and GGPP, respectively. Bisphosphonates (BPs), the inhibitors of FPPS, constitute one of the main classes of drugs used to treat bone-associated diseases. In retrospective cohort studies, the exposure to BPs (alendronate, risedronate) was associated with reduced T2D incidence.^12^ Moreover, the administration of BPs was shown to positively affect diabetes-related indices, insulin, fasting plasma glucose (FPG), and hemoglobin A1c (HbA1c).^13^ On the other hand, overexpression of muscle,^14^ adipose,^15^ and liver^16^ GGPPS may contribute to insulin resistance pathogenesis. Therefore, inhibition of FPPS and GGPPS may be considered a strategy for insulin resistance treatment. However, additional large-scale trials are needed to verify these relationships.

The mechanisms by which statins and bisphosphonate treatments induce or bypass T2D are not fully understood. It is accepted that their pleiotropic effects might result from changes occurring downstream from these enzymes and that small GTPases are implicated here. Small GTPases are regulated by several protein–protein interactions (PPIs) and PTMs. One of the most studied PTMs is protein prenylation, which is crucial for glucose-stimulated insulin secretion (GSIS) by pancreatic β-cells.^17^ However, several proteins within the mevalonate pathway may be implicated in T2D development. Here, we discuss the mechanisms of small GTPase prenylation and how inhibition of various points in the mevalonate pathway might affect protein prenylation and functioning of pancreas and liver, skeletal muscle, kidneys, adipose tissue, and contribute to chronic inflammation involved in T2D development.

## 2 Overview of Superfamily of Small GTPases and Enzymes within the Mevalonate Pathway

The human Ras superfamily of small GTPases, including over 150 proteins, comprises five major subfamilies: Ras, Rab, Rho, Ran, and Arf. Six major subgroups (Ras, Ral, Rap, Rad, Rheb, and Rit) have been identified within the Ras subfamily, which includes 36 human members. The Ras branch regulates cell proliferation, differentiation, and survival.^18^ With over 60 members in humans, Rab proteins (Ras-related in the brain) form the largest subgroup of the small GTPase superfamily with the principal function of coordinating the transport of proteins and membranes between organelles. Twenty-two genes in humans encode 20 Rho GTPases (Ras homologue) distributed into eight subfamilies (Rac, Cdc42, Rho, RhoD/RhoF, RhoH, RhoU/RhoV, RhoBTB, and Rnd). The Rho family members are essential coordinators of the actin filament network, synchronizing cell shape and movement with intercellular communication, propagation, and differentiation.^19^ The single Ran (Ras-related nuclear protein) is one-of-a-kind among other GTPases due to its acidic tail at the C-terminus and the lack of the CAAX motif that precludes attachment to lipid membranes. Ran regulates the transport of molecules between the nucleus and cytoplasm and controls cell cycle progression. The adenosine diphosphate-ribosylation factor (Arf) family comprises 29 members in humans and includes Arf isoforms, Arf-like proteins (Arl), and Sar1 proteins. Arf family lacks the C-terminal prenylation signal. Many of Arf family members are myristoylated at the N-terminus for membrane targeting and control vesicular trafficking, motility, division, apoptosis, and transcriptional regulation.^18^

**Figure 1:**
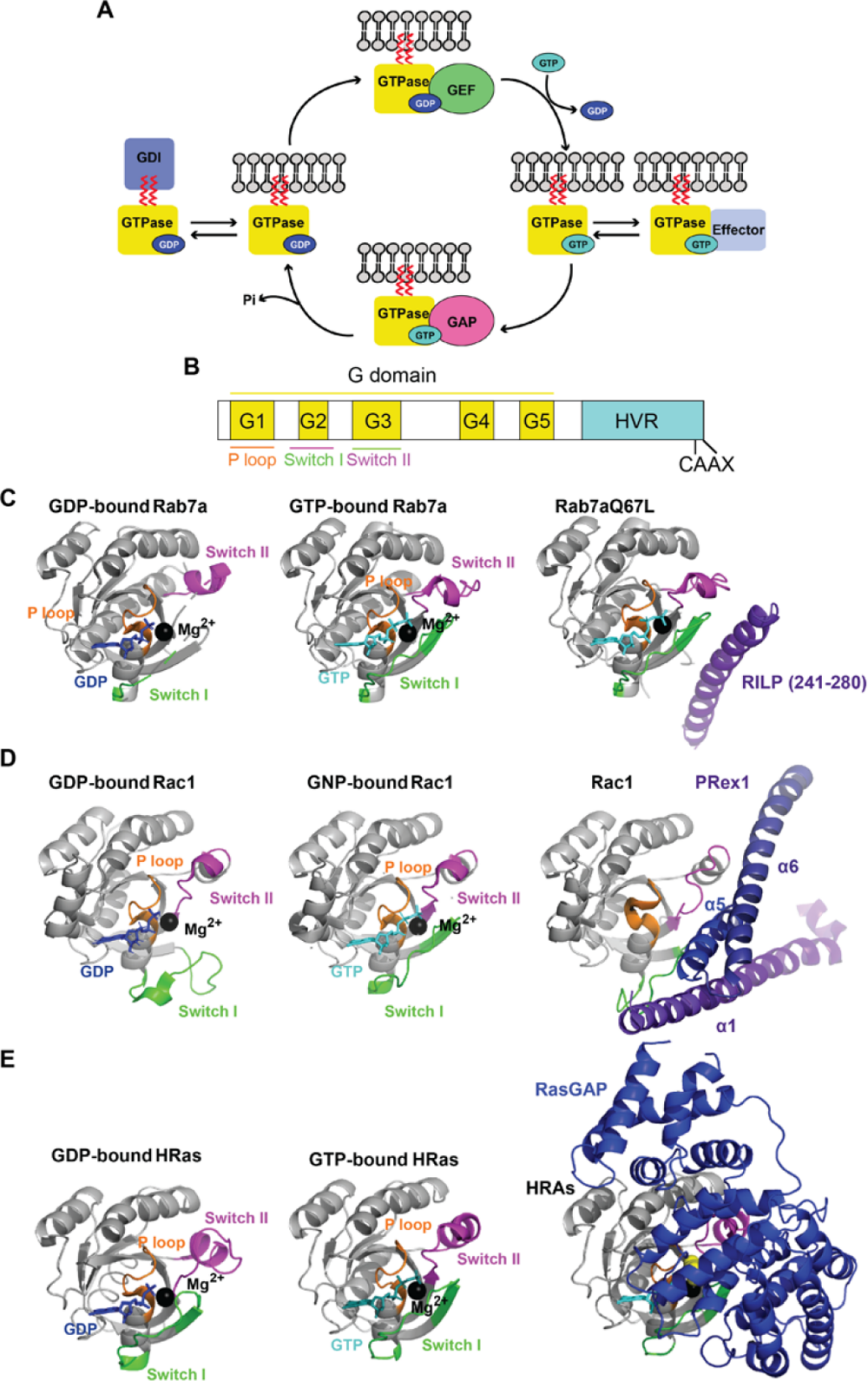
Small GTPase cycle: (A) Interaction with GEF mediates the exchange of GDP for GTP, allows activation, interaction with effectors, and initiation of the signal cascade. Interaction with GAP increases GTP hydrolysis, leading to G protein deactivation. Interaction with GDI keeps small GTPase in an off-state and prevents membrane localization. (B) The conserved architecture of the G domain present in small GTPases (for sequence alignment of Rab, Rho and Ras GTPases implicated in diabetes, see Supplementary Figure S1). (C) Crystal structures of Rab7a: left, inactivated (GDP-bound, PDB; 1VG1); middle, activated (GTP-bound, PDB: 1VG8); right, with its effector RILP (PDB: 1YHN, only part of RILP interacting with Rab7a is shown). (D) Crystal structures of Rac1: left, inactivated (GDP-bound, PDB: 6AGP), middle, activated (GNP-bound, PDB: 3TH5); right, with its effector PRex1 (PDB: 4YON, only domains of PREx1 interacting with Rac1(a1, a5, and a6) are shown). (E) Crystal structures of HRas: left, inactivated (GDP-bound, PDB: 4Q21); middle, activated (GTP-bound; PDB: 1QRA); right, with RasGAP (PDB: 1WQ1). The P loop is represented in orange, switch I in green, switch II in magenta, coordinated magnesium ion in black, GDP in dark blue, and GTP or GTP analogues in cyan. GNP: phosphoaminophosphonic acid-guanylate ester nonhydrolyzable GTP analogue. The corresponding Supplementary Table 1 contains the list of PDB codes for mammalian small GTPases implicated in diabetes, in GDP and GTP-bound form, with effector/GEF/GAP, when available.

Small GTPases are guanine nucleotide-dependent molecular switches, active when in complex with GTP and inactive when in complex with GDP. Active small G proteins recruit effectors to the membranes and trigger signal cascades. It requires a tight regulation and small GTPases have three types of controllers, the GTPase-activating proteins (GAPs), the guanine nucleotide exchange factors (GEFs), and the guanine nucleotide dissociation inhibitors (GDIs). GEFs are positive regulators by promoting GDP dissociation, while GAPs are negative regulators by binding to the GTPase and enhancing hydrolysis of GTP. In the case of Rho and Rab, GDIs perturb GAP and GEF regulation and mask the prenyl moiety, thus preventing the association with target membranes (Figure 1A).^18^ Abnormal activity of some regulatory proteins is linked to diabetic conditions, *e.g.*, dysregulated production of GDI2 contributes to IR.^20^

Members of the small GTPases share a conserved G domain composed of five loops (G1–G5) that are capable of GTP binding and hydrolysis (Figure 1B, in yellow). The G1 motif (P-loop, Figure 1B, in orange) binds the phosphate groups of GTP and GDP, the G2 motif (switch I, Figure 1B, in green) involved in coordinating of Mg^2+^ ion with the β- and γ-phosphate is a site for effector and GAP attachment (Figure 1E: HRas-RasGAP; Supplementary Table 1), the G3 motif (switch II, Figure 1B, in magenta) activates a catalytic water molecule for GTP to GDP hydrolysis, the G4 motif provides hydrogen bonds with guanine rings, and the G5 region interacts with guanine via water-mediated hydrogen bonds. Upon exchange of GDP to GTP, effector binding is governed by switch I and switch II, very flexible regions, for which the dynamics differ depending on whether GTP or GDP is attached (Figure 1C–E; Supplementary Table 1). The additional C-terminal hypervariable region (HVR), which accommodates a polybasic region (PBR) and cysteines, regulates GTPase association with target membranes (Figure 1B, Supplementary Figure S1).^18^

Small G proteins regulate various effectors (Table 1). GTP binding energy is used to stabilize the switch I and II regions, required for effector recognition (Figure 1C: Rab7a-RILP, 1D: Rac1-PRex1). GTP hydrolysis induces conformational change and a flexibility in the region interacting with the effector. The binding of some effectors slows down GTP hydrolysis, while interaction with GAPs speeds it up.^18^

**Table 1:** Small GTPases Involved in Insulin Release from Pancreatic β-Cells under Physiological Conditions

GTPase	localization	interacting protein	function	refs
**Rab GTPases**				
Rab1a	ER-Golgi membranes	Golgin-84	conversion of proinsulin to insulin; maintaining Golgi stability	Liu et al.^32^
Rab2a	ERGIC	GAPDH	vesicular transport of proinsulin from ERGIC to the Golgi; a switch protein that facilitates ER-associated degradation or secretion of (pro)insulin	Sugawara et al.^33^
		Noc2	ternary Rab2a-Noc2-Rab27a complex mediates processing proinsulin to insulin	Matsunaga et al.^34^
Rab3	ISG	RIMs	Rim2α–Rab3a interaction is required for the docking of insulin granule	Yasuda et al.^35^
		granuphilin	granuphilin-Rab3a augments insulin granule exocytosis	Coppola et al.^36^
		Noc2	Noc2-Rab3 positively regulates insulin secretion required for maintenance of RRP	Matsumoto et al.^37^
			all Rab3, except for Rab3c, are required for Ca^2+^-dependent insulun secretion	Cazares et al.^38^
Rab7	late endosomes, lysosomes	RILP	insulin secretion is inhibited by RILP, which controls lysosomal degradation of proinsulin by interacting with lysosome-located Rab7	Zhou et al.^39^
Rab8a	PM, ISG		regulation of Kir6.2 membrane trafficking	Uchida et al.^40^
Rab11b	ISG	Rip11	cAMP (but not glucose)-induced insulin release by modulating the recycling of the proteins associated with the exocytotic back to immature granules	Sugawara et al.^41^
Rab26	ISG	RILP	insulin secretion is inhibited by RILP, which controls lysosomal degradation of proinsulin	Zhou et al.^39^
Rab27a	ISG		defines the total quantity of RP and RRP	Cazares et al.^38^
		granuphilin	granuphilin forms a regulated Rab27a complex with Munc18-1 and Syntaxin1a, regulates docking of insulin granules, and inhibits subsequent fusion of docked granules	Yi et al.^42^
				Torii et al.^43^
		exophilin-7	movement of the granule along the actin filament	Wang et al.^44^
		exophilin-8	tripartite complex of exophilin-8, Rab27a, and myosin Va mediates the fusion of undocked granules with the cell surface phospholipids	Mizuno et al.^45^
		Noc2	Noc2–Rab27a complex on peripheral mature granules mediates vesicle priming and insulin exocytosis	Matsunaga et al.^34^
		coronin 3	Rab27a–GDP–coronin 3, in complex with IQGAP1, is crucial for endocytosis of insulin granules	Kimura et al.^46^
Rab37	ISG		final steps of insulin exocytosis	Ljubicic et al.^47^
**Rho GTPases**				
RhoA	PM	ROCK	actin cytoskeleton stabilization and GSIS inhibition	Hammar et al.^48^
Cdc42	cytosol, ISG, PM	N-WASP	N-WASP binds Cdc42 to actin via the Arp2/3 complex necessary for GSIS	Uenishi et al.^49^
		PAK-1	F-actin remodeling and granule recruitment to the plasma membrane during the first phase of insulin release	Wang et al.,^50^ Kalwat et al.^51^
		syntaxin 1, syntaxin 4, VAMP2	Cdc42 and VAMP2 form heterotrimeric complexes with syntaxin 1 and 4	Nevins et al.,^52^ Daniel et al.^53^
		caveolin-1	caveolin-1 binds to Cdc42 present on ISG. The complex translocates to the plasma membrane and dissociates	Nevins et al.^54^
		coronin 3, IQGAP1	endocytosis of the insulin secretory membrane requires a complex containing IQGAP1, GDP-bound Rab27a, and coronin 3.	Kimura et al.^55^
Rac1	cytosol, PM		insulin secretion via depolymerization of F-actin	Asahara et al.^56^
		PAK1	glucose-induced Rac1-mediated F-actin remodeling and insulin secretion	Kalwat et al.^51^
		Tiam1 (GEF)	modulation of Tiam1/Rac1-dependent signaling step in GSIS	Veluthakal et al.^17^
		Vav2	Vav2-Rac1 required for glucose-induced actin depolymerization and GSIS	Veluthakal et al.^57^
		P-Rex1 (GEF)	initiates the cascade of events leading to GSIS	Thamilselvan et al.^58^
		Trio (GEF)	rearrangement of Rac1 to the cell surface required for GSIS	Dufurrena et al.
		Kalirin (GEF)	rearrangement of Rac1 to the cell surface required for GSIS	Dufurrena et al.
**Ras GTPases**				
Rap1	PM	Epac2 (GEF)	Epac2, a cAMP binding protein, regulates insulin exocytosis	Shibasaki et al.^59^
RalA	PM, ISG	RalGDS	modulates the dynamics of the actin cytoskeleton	Ljubicic et al.^60^
		Sec6	tethers secretory granules through its regulated association with the exocyst (Sec6) complex	Lopez et al.^61^
		Ca_v_α_2_δ-1 subunit of VDCC	RalA binds α_2_δ-1 on insulin granules to tether granules to plasma membrane Ca^2+^channels (a step to prepare for the assembly of excitosome and exocyst complexes required for biphasic insulin secretion)	Xie et al.^62^

**Figure 2:**
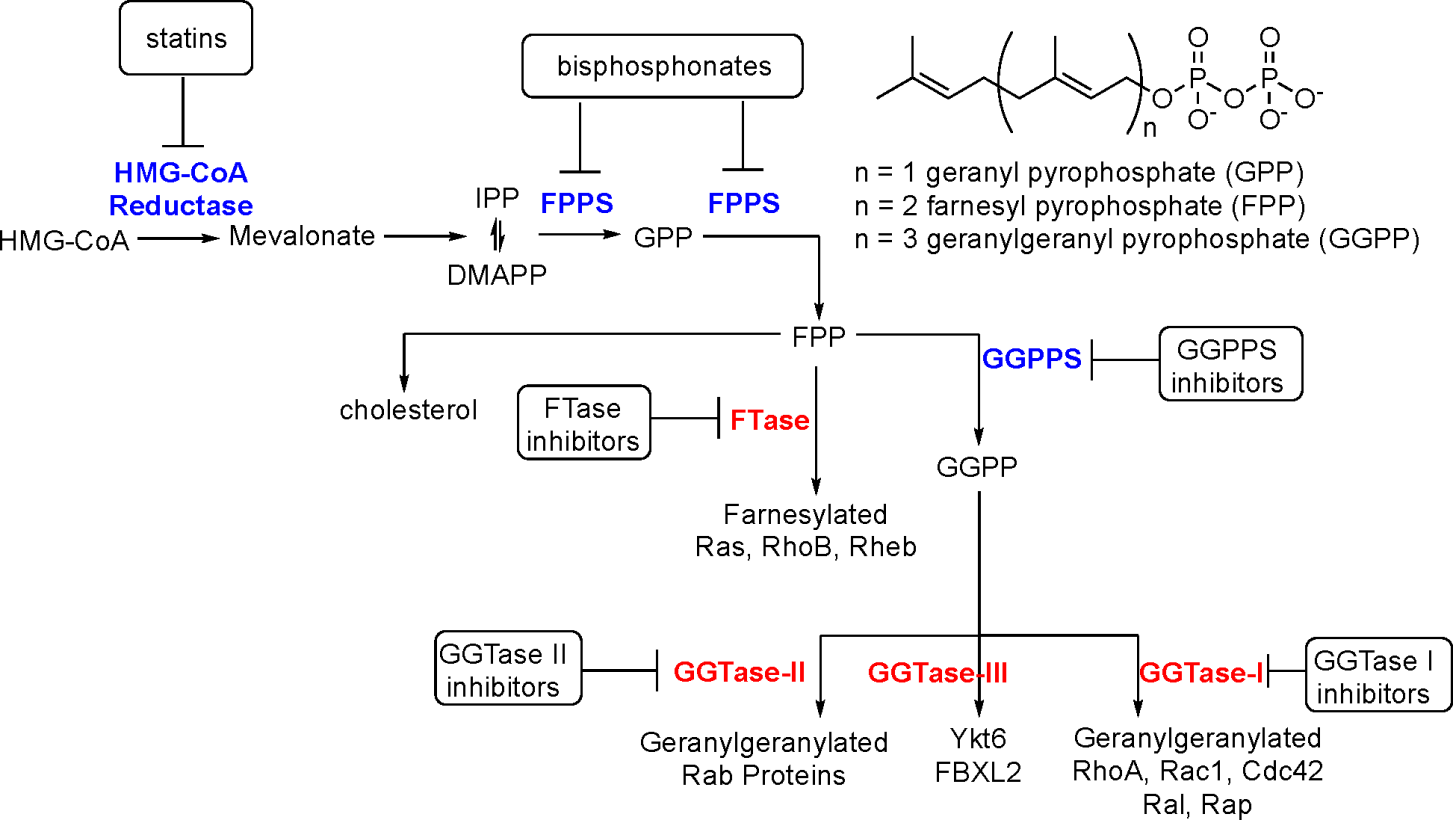
Schematic representation of mevalonate pathway. HMG-CoA reductase catalyzes the formation of mevalonate from HMG-CoA. FPPS mediates further conversion to GPP and FPP. FTase catalyzes attachment of FPP to Ras, Rho, and Rheb proteins (in the process called farnesylation). GGPPS catalyzes the conversion of FPP to GGPP that can be post-translationally added to RhoA, RAc1, Cd42, Ral, and Rap by GGTase-I, Rab proteins by GGTase-II, and Ykt6 and FBXL2 by GGTase-III.

Besides GDP/GTP binding, small GTPases usually carry a post-translationally attached prenyl tail at cysteine residues present in or located close to the CAAX motif. For that purpose, the farnesyl and geranylgeranyl chains are added to GTPases, and the substrates, FPP and GGPP, are synthesized via the mevalonate pathway (Figure 2). The mevalonate pathway is an essential biosynthetic step that produces components for the cholesterol biosynthesis or FPP and GGPP, and it starts from the condensation of the monomers, isopentenyl diphosphate (IPP) with its isomer, dimethylallyl pyrophosphate (DMAPP).^21^

**Figure 3:**
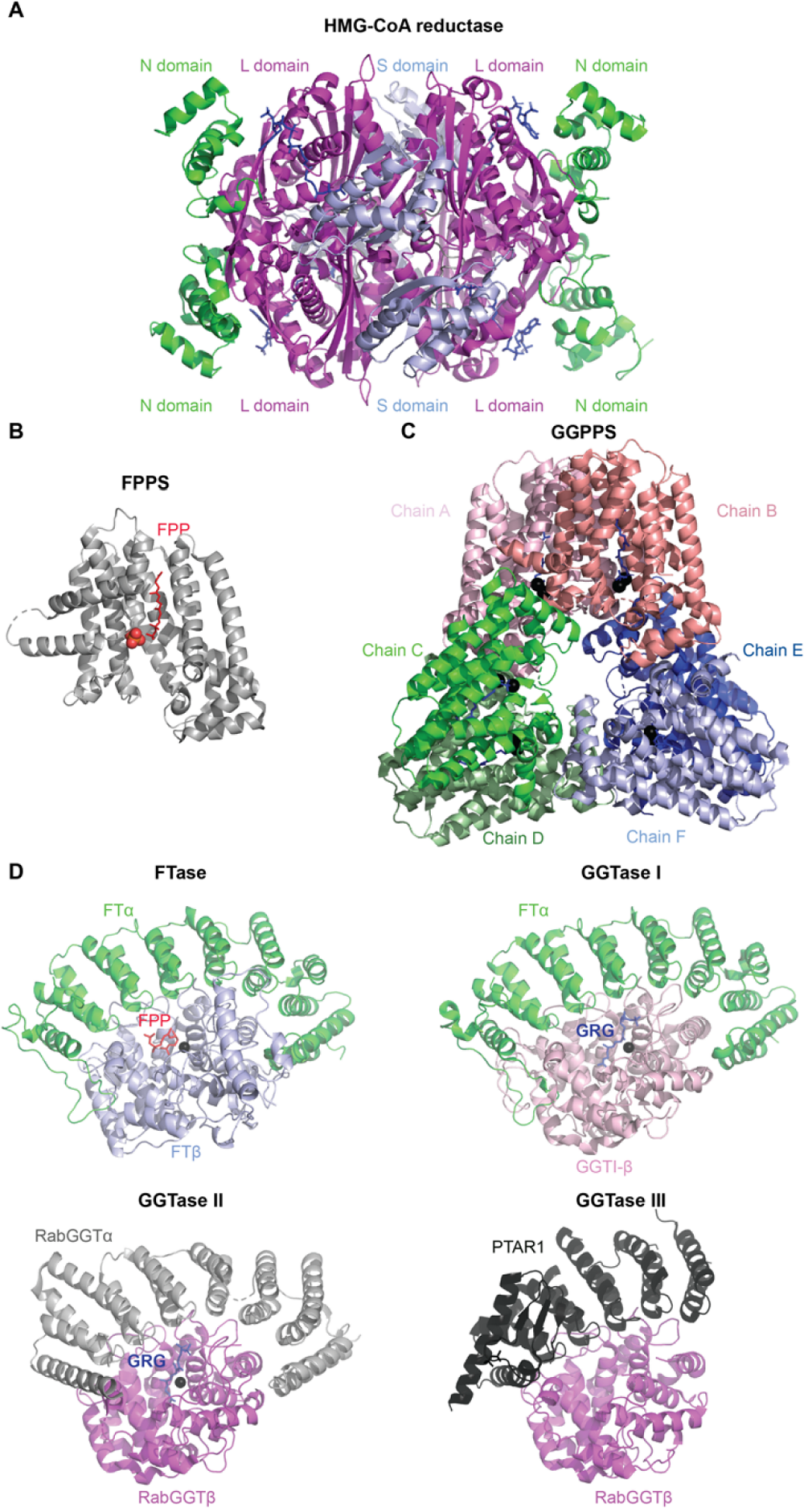
Structural overview of enzymes within the mevalonate pathway and prenyltransferases. (A) HMG-CoA reductase (PDB: 1DQ9) is a homotetramer. Each subunit comprises an N domain (in green), large L domains (in magenta), and an S domain (in light blue). (B) FPPS (PDB: 5JA0) PO_4_ in red. (C) GGPPS (PDB: 2Q80) is a hexameter composed of three dimers: chain A–B (in pink), chain C–D (in green), and chain E–F (in blue). Mg^2+^ is represented in black, and GRG in dark blue. (D) Comparison of structures of prenyltransferases: FTase (PDB: 1FPP), GGTase-I (PDB: 1N4P), GGTase-II (PDB: 3DST), and GGTase-III (PDB: 6J6X). The α and β subunits are color-coded, and the shared domains have the same color. Zn^2+^ is presented in black. The corresponding Supplementary Table S2 contains the list of PDB codes for mammalian enzymes within the mevalonate pathway and prenyltransferases implicated in diabetes, in GDP and GTP-bound form, with substrate/product/inhibitor, when available.

HMG-CoA reductase produces mevalonate in the rate-limiting step in the pathway. Mammalian HMG-CoA reductase functions as a homotetramer (Figure 3A; Supplementary Table 2). Each monomer consists of the cytosolic C-terminal catalytic domain, the L domain responsible for substrate binding, the S domain binding NADPH, and the N-terminal segment for anchoring to the ER membrane. Statins bind stronger to the L domain than HMG-CoA, *e.g.*, with the inhibitory concentration values of 3.8–6.2 nM for atorvastatin.^22^

FPPS catalyzes the synthesis of 10-carbon geranyl pyrophosphate (GPP) and the 15-carbon FPP, whereas GGPPS synthesizes the 20-carbon GGPP. Even though free GPP has been detected in cultured human cells,^23^ as far as we know, the geranylated entities have not been detected in human cells yet. The majority of the studies on protein prenylation concentrate on farnesylated and geranylgeranylated proteins and developing the suitable tools.^24^

Although human FPPS exists as a homodimer (Figure 3B; Supplementary Table 2), human GGPPS is a hexamer assembled from three dimers (Figure 3C; Supplementary Table 2). Despite low sequence identity, both isoprenoid synthases adopt a similar all α-helical structure. At least three small-molecule binding sites are present in the structure of FFPS, namely, allosteric pocket, allylic substrate (DMAPP and GPP) binding site, and homoallylic substrate (IPP) binding site, with the latter two having high similarity to those found in FPPS. The product inhibitor pocket has been identified in GGPPS as well.^21^

FPP and GGPP moieties are utilized by four distinct prenyltransferases, namely, farnesyltransferase (FTase), geranylgeranyltransferase I (GGTase-I), Rab geranylgeranyl transferase (GGTase-II/RGGT), and geranylgeranyltransferase III (GGTase-III). All enzymes catalyze the formation of the thioether linkage with the Cys residue located in the prenylation recognition sequence at the C terminus of selected proteins. FTase and GGTase-I transfer a respective prenyl group to protein substrates containing carboxyl-terminal CAAX motifs where C is cysteine, A is aliphatic, and X is any residue. Usually, FTase prefers Cys, Ser, Met, Ala, or Gln while GGTase-I selects Leu, Ile, or Phe at the X position.^25^ Ras, RhoB, and Rheb have been identified as substrates of FTase while GTPases geranylgeranylated by GGTase-I include Rho, Ral, and Rap. There are examples when a protein is either farnesylated or geranylgeranylated, for instance, RhoB. On the other hand, in the case of K-Ras, inhibition of FTase was linked to a compensatory GGTase-I upregulation that can be a reason for the insufficient clinical efficacy of anticancer FTase inhibitors. Therefore, dual FTase/GGTase-I inhibitors may prove a more effective therapeutic approach.^26^

GGTase-II (Rab geranylgeranyl transferase; RGGT) exclusively geranylgeranylates C-terminally localized CXC and CC motifs in Rab family members. Unlike FTase and GGTase-I, prenylation of Rab proteins by RGGT must be associated with REP1/2 chaperone proteins (Rab escort protein 1/2). Most Rab proteins are doubly geranylgeranylated in a sequential fashion without dissociation of the monoprenyl intermediate.^25^

The fourth type of protein prenyltransferase, GGTase-III, has been discovered very recently. This enzyme catalyzes the double prenylation of the FBXL2 ubiquitin ligase and Golgi SNARE protein Ykt6 in collaboration with FTase. Chaperone SKP1 protein is required for geranylgeranylation by GGTase-III.^27,28^ According to the authors’ knowledge, no inhibitors of this enzyme have been reported yet.

Each prenyltransferase exists as a heterodimer with the active site formed at these proteins’ interface and made up of α- and β-subunits (Figure 3D; Supplementary Table 2). FTase and GGTase-I have different catalytic β-subunits (FNTB/FTβ and GGT1β, respectively) and share a common α-subunit (FNTA/FTα). In turn, RGGT and GGTase-III share identical β subunit (RABGGTβ) but contain distinct α subunits (RABGGTα and PTAR1, respectively). The RABGGTβ subunit of RGGT and GGTase-III is probably necessary for double prenylation due to its hydrophobic tunnel structure.^28^

All protein prenyltransferases are metalloenzymes. A Zn^2+^ ion (a thiolate) is bound by the catalytic domain of the β subunit of GGTases. Additionally, FTase requires Mg^2+^ that stabilizes PPi leaving group of FPP.

## 3 Small GTPases as Regulators of the Insulin Trafficking and Exocytosis in Pancreatic β-Cells

Small GTPases are critical in maintaining whole-body glucose homeostasis acting predominantly in metabolically active tissues, including the pancreas, skeletal muscles, liver and adipocytes. The pancreas plays a key role in this network by secreting the blood-glucose-lowering hormone insulin, produced by β-cells located within islets of Langerhans. Preproinsulin is synthesized on the cytoplasmic side of the ER and translocated to the ER, where the signal peptide is cleaved. The resulting proinsulin is transported to the *cis*-face of the Golgi apparatus and starts to be packaged after reaching Trans-Golgi Network (TGN). Proteolytic cleavage of proinsulin results in the formation of insulin. Insulin crystallizes with zinc and calcium in the form of dense-core granules during the granule maturation process. The readily releasable pools (RRP) and the reserved pool are two intracellular pools of dense-core insulin granules. When blood glucose level is low, the actin cytoskeleton prevents insulin secretory granules (ISGs) from reaching their release sites.^29^

**Figure 4:**
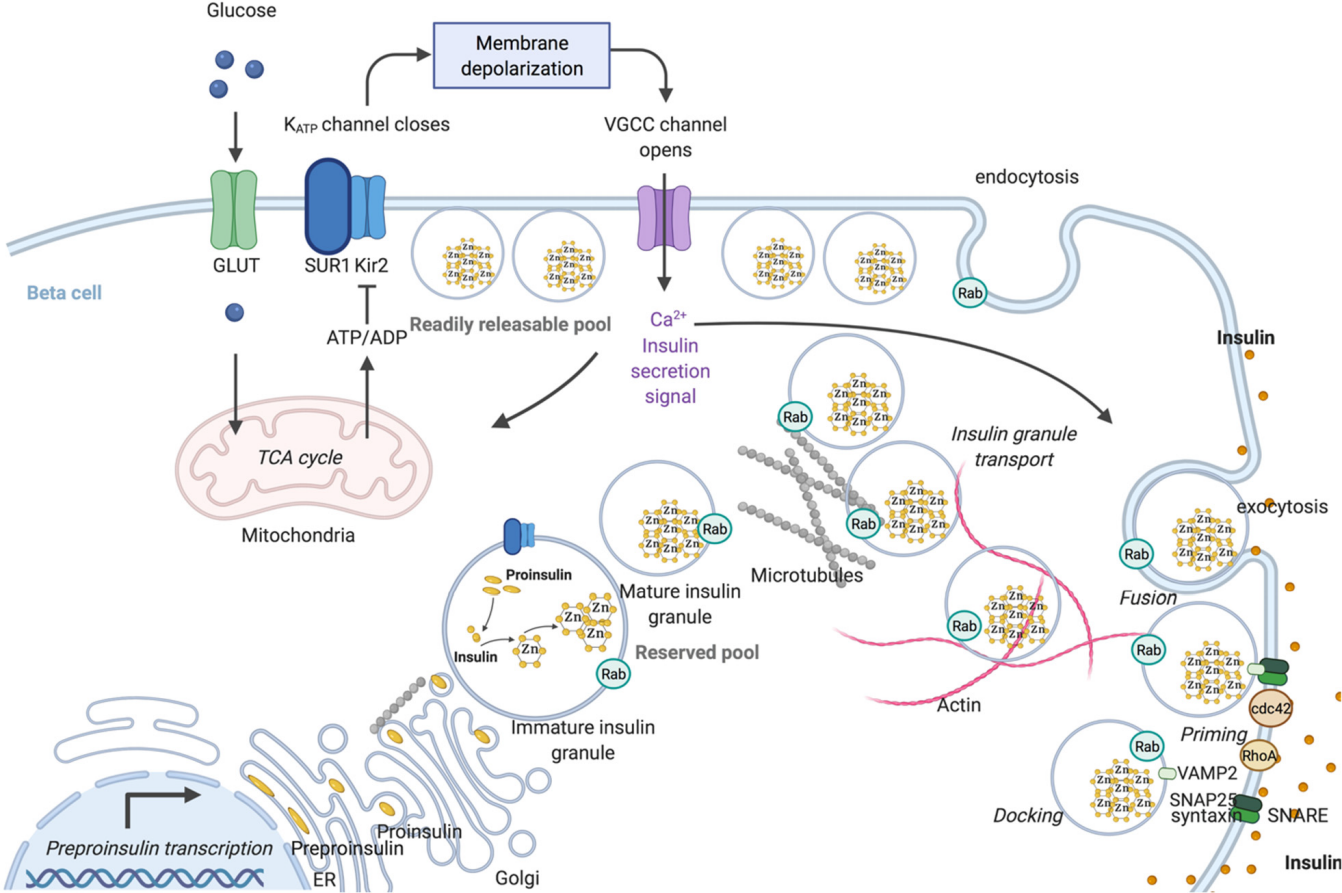
Schematic representation of insulin synthesis and trafficking and exocytosis of insulin containing granules (created in BioRender.com). Proinsulin processing occurs in the lumen of ER and insulin is stored as a hexamer in complex with Zn^2+^. Glucose enters the cells and via mitochondrial ATP synthesis raises the ATP-to-ADP ratio, causing the ATP-sensitive K^+^ (KATP) channels to close. Following cellular depolarization, VGGC is activated, causing extracellular Ca^2+^ influx and insulin granule fusion with the plasma membrane. Specific sets of Rab GTPases regulate insulin secretory granule transport, endocytosis, and the three main stages of insulin granule exocytosis (docking, priming, and fusion). For the sake of simplicity, we have not included all the specific Rabs involved that have been described in Table 1.

When plasma glucose levels are high in humans, glucose enters the β-cells, primarily through the cell membrane glucose transporters GLUT1 and GLUT3, although GLUT2 expression was also demonstrated by several groups.^30^ Upon uptake, glucose is metabolized and a high ATP-to-ADP ratio triggers membrane depolarization by closing ATP-dependent potassium channels (K_ATP_). Consequently, voltage-gated calcium channels (VGCC) open and that results in calcium influx, which induces docking and fusion with the plasma membrane (exocytosis of insulin granule). The docking and fusion of insulin granules are orchestrated by the soluble *N*-ethylmaleimide sensitive factor attachment receptor (SNARE) complex. The target-localized (t-SNARE) proteins in the cell surface (SNAP25 and Syntaxin) interact with VAMP (vesicle-associated membrane protein, v-SNARE) on the insulin granules (Figure 4). Under high glucose, the actin cytoskeleton is reorganized, allowing them to move to the plasma membrane. Such glucose-mediated exocytosis of different functional granule pools occurs in response to elevated glucose concentration in a biphasic manner. The rapid first phase (usually the first 10 min) results from fusion and secretion of a subset of plasma membrane-docked granules that are primed with a fully assembled exocytosis machinery (RRP). F-actin filaments are important for the short-range movement of RRP. The second step entails the recruitment of granules from the inside of the cell and microtubule transport.^29^

The trafficking of the insulin granules is controlled by several Ras family GTPases and their effectors. Various Rab proteins are associated with the secretory granules and regulate the transport, priming, docking, and fusion of ISGs at the plasma membrane (Figure 3 and Table 1). For example, Rab3 allows ISG docking and tethering at the correct target membrane by interacting with RIM2α and the clustering of the SNARE Syntaxin1 and its binding partner munc18-1. In turn, the Rho family, including Cdc42, Rac, and RhoA, is instrumental in insulin secretion via F-actin remodeling and vesicle fusion regulation. Cdc42 was also shown to be crucial for endocytosis of insulin vesicles. Rap1 and RalA, although less studied, also elicit regulatory effects in insulin release.^19,29^ The detailed information on specific functions of small G proteins in insulin secretion by pancreatic β-cells is summarized in Table 1.

Most small GTPases involved in insulin trafficking and secretion are required to be prenylated to function for their biological role and interaction with their respective effectors. FTase, GGTase-I, and GGTase-II are expressed in β-cell lines and pancreatic islets. Studies utilizing inhibitors of HMG-CoA reductase (atorvastatin, lovastatin, simvastatin), GGPPS (digeranyl bisphosphonate), FTase (FTI-277, FTI-2628, allyl- or vinyl-farnesols, limonene, manumycin, perillic acid), and GGTase-I (GGTI-298, GGTI-2133, GGTI-2147; GGTI-2368, allyl- or vinyl- geraniols) as well as siRNA-mediated silencing of *Rggta* and *Rggtb* revealed that prenylation of small GTPases is essential for β-cell function and insulin secretion.^31^

## 4 Small GTPases as Regulators of GLUT4 Trafficking

Insulin-stimulated glucose uptake into skeletal muscle cells and adipocytes assumes a central role in glucose homeostasis in the body. Most (80–90%) of the infused glucose is absorbed by skeletal muscles that store glucose as glycogen and utilize it in glycolysis; however, adipocytes also exert a critical control in the regulation of blood glucose levels. Insulin promotes the exocytosis of intracellular vesicles containing GLUT4 glucose transporters, the most abundant glucose transporter in muscle and fat cells. In the basal state, GLUT4 locates intracellularly in endosomes, TGN, specialized perinuclear glucose transporter storage vesicles (GSVs), and more peripheral insulin-responsive vesicles (IRVs).^63^

**Figure 5:**
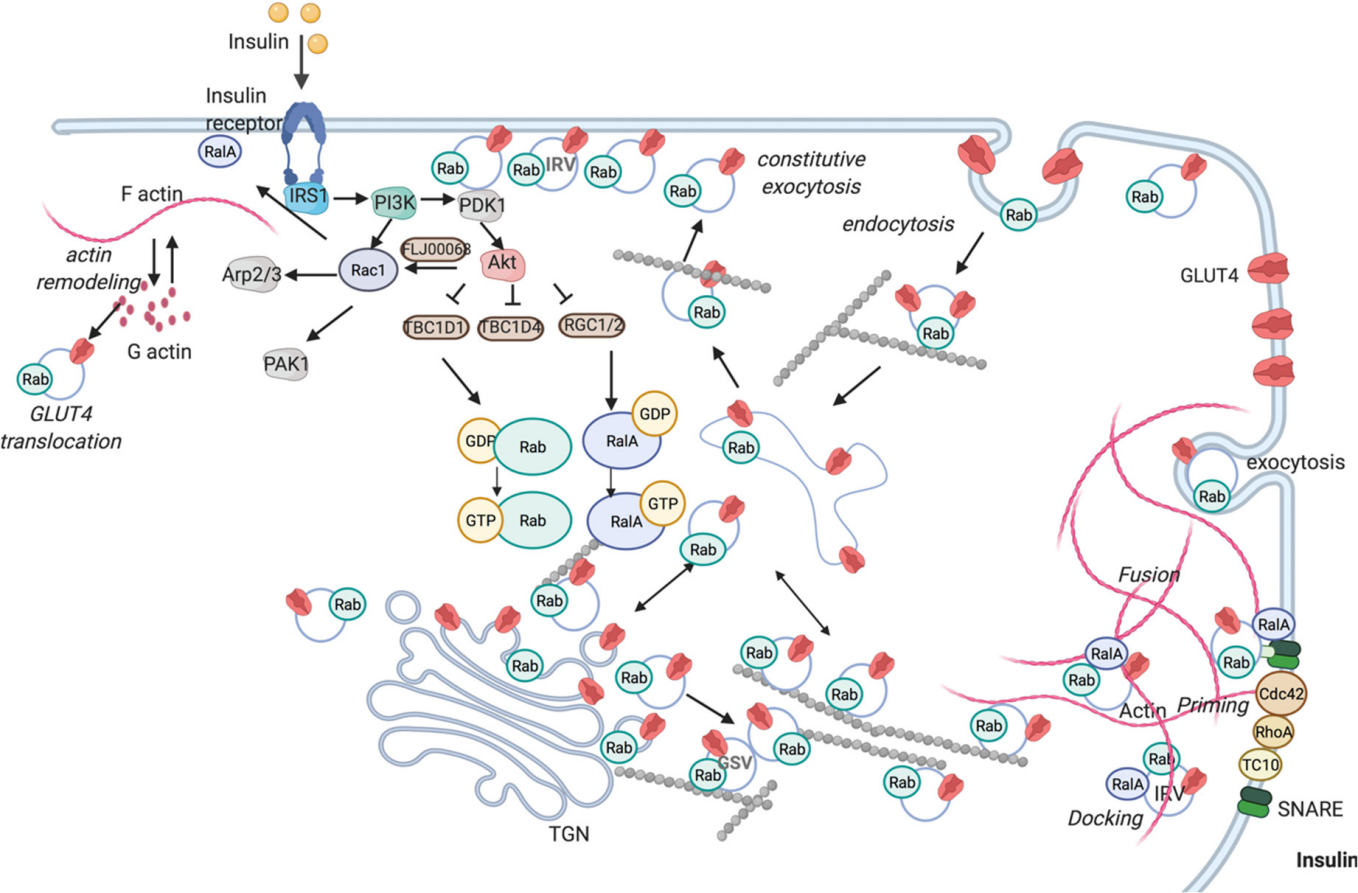
Scheme of the insulin-regulated transport of GLUT4 vesicles translocation and exocytosis (created in BioRender.com). Insulin binds the insulin receptor that induces the translocation of GLUT4 storage vesicles by activating the PI3K signaling cascade. PI3K catalyzes the formation of phosphatidylinositol (3,4,5) trisphosphate leading to the action of PDK1, which in turn stimulates Akt. Activated Akt phosphorylates and inactivates GAPs (*e.g.*, TBC1D1, TBC1D4, RGC1/2). GAPs inhibition shifts small GTPases from the GDP- to a more active GTP-loaded state. Rac1 facilitates GLUT4 plasma membrane association via actin filament remodeling. GTP-loaded Rabs and other Ras superfamily members permit GLUT4 storage vesicle translocation to the cell surface for fusion. In addition to the main PI3K pathway, the Rho family GTPases (*e.g.*, RhoA, Cdc42, TC10) mediate insulin signaling in regulating GLUT4 translocation. For the sake of clarity, we have not included all the specific Rabs involved that have been described in Table 2.

The insulin binding to the tyrosine kinase receptor activates its autophosphorylation and initiates a signaling cascade starting from phosphorylation of insulin receptor substrates (IRS1 and IRS2). IRS, in turn, phosphorylates phosphatidyl inositol-3-kinase (PI3K) and promotes downstream signaling. PI3K constitutes a branch point in insulin signaling activating Akt and Rac1, which in parallel promote GLUT4 transport to the plasma membrane, permitting glucose intake.^64^ Akt phosphorylates various GAPs (*e.g.*, TBC1D1, TBC1D4), reducing the inactivation of their cognate GTPases (Figure 5). Several Rab GTPases, including Rab4, Rab5, Rab7, Rab8a, Rab10, Rab11, Rab13, Rab14, Rab28, and Rab35, with effector proteins were demonstrated to confer directionality to GLUT4 vesicle traffic. Insulin also activates Rho and Ras GTPases mainly affecting actin remodeling (Table 2). Glucose uptake by GLUT4 also occurs upon muscle contraction; however, muscle contraction and insulin target separate GLUT4 pools. During muscle contraction, the AMP/ATP ratio increases, leading to activation of AMP-activated protein kinase (AMPK), the cellular energy sensor. AMPK, in turn, phosphorylates TBC1D1 and TBC1D4 activating target Rabs.^65^ Rac1 acts as another contributor to contraction-stimulated glucose transport mediating the stretch-sensitive component.^66^

**Table 2:** Small GTPases Involved in Insulin-Induced GLUT4 Translocation

GTPase	localization	interacting protein	function	refs
***adipocytes***				
**Rab GTPases**				
Rab4a, Rab4b	IRV	syntaxin 4	involvement in GSV sorting and fusion	Li et al.^67^
			recycling of GLUT4 via endosomes	Chen et al.^68^
Rab5a	early endosomes	dynein	insulin signaling deactivates Rab5 and impedes dynein microtubule interaction, slowing GLUT4 inward movement	Tessneer et al.^69^
Rab8a	endosomes, TGN, GSV	TBC1D4 (GAP)	GLUT4 translocation; cell surface endosome cycling of GLUT4	Mîinea et al.,^70^ Chen et al.^68^
		MyoVa	insulin-mediated signaling augments Rab8a–MyoVa interaction to drive GLUT4-containing vesicles to the cell surface.	Sun et al.^71^
Rab10	perinuclear endosome/TGN, GSV	TBC1D4 (GAP)	accumulation of GLUT4-containing vesicles at the cell surface	Mîinea et al.,^70^ Sadacca et al.^72^
		MyoVa	Rab10–MyoVa interaction facilitates the transport of GSVs and docking at the cell surface.	Chen et al.^68^
		SEC16A	SEC16A–Rab10 interaction promotes GLUT4 mobilization from the intracellular compartments to the cell to accelerate formation of the GSV	Bruno et al.^73^
		Exoc6/6b	Rab10-Exoc6/6b promotes the fusion of GLUT4-containing vesicles with the cell surface	Sano et al.^74^
		Exoc7	Exoc7 exerts a critical function in insulin-stimulated GLUT4 exocytosis	Wang et al.^75^
		Rlf (GEF)	Rab10 promotes RalA activation by recruiting Rlf.	Karunanithi et al.^76^
		RABIF (GEF)	RABIF enhances Rab10 stability and GLUT4 exocytosis	Gulbranson et al.^77^
		Dennd4C (GEF)	primary GEF required for GLUT4 translocation	Sano et al.^78^
Rab11	Golgi, endosomes	Rip11	Rip11 is a scaffolding protein in the coupling of GLUT4-containing vesicles with the cell surface	Welsh et al.^79^
			GLUT4 transport from the endosomal compartments to GSV	Zeigerer et al.^80^
Rab14	TGN, endosomes, GSV		GLUT4 transport to the plasma membrane via transferrin receptor-positive endosomal structures.	Chen et al.^68^
			early endosomes-to-TGN transport of GLUT4	Reed et al.^81^
			Rab14 is a controller of GLUT4 sorting into vesicles (upstream of Rab10)	Sadacca et al.^72^
		TBC1D4 (GAP)	GLUT4 sorting into GSV	Mîinea et al.^70^
Rab28		TBC1D1 (GAP), TBC1D4 (GAP)	GLUT4 trafficking	Zhou et al.^82^
Rab35	PM	TBC1D13 (GAP)	GLUT4 translocation (a trafficking pathway from early endosomes)	Davey et al.^83^
**Rho GTPases**				
TC10	lipid rafts in PM	CIP4/2	GLUT4 trafficking, docking, and fusion with the cell surface	Chang et al.^84^
		N-WASP	N-WASP-Arp2/3 is required to mobilize cortical F-actin and GLUT4 translocation	Jiang et al.^85^
RhoA	PM		RhoA regulates glucose transport via remodeling of actin cytoskeleton	Duong and Chun^86^
			RhoA modulates the activity of IRS-1	Takaguri et al.^87^
		ROCK1	GLUT4 translocation and actin cytoskeleton remodeling	Chun et al.^88^
Cdc42	perinuclear cytosol, PM		GLUT4 translocation and glucose transport	Usui et al.^89^
Rac1	cytosol, PM	P-Rex1 (GEF)	P-Rex1-facilitated GLUT4 plasma membrane association via regulation of the actin cytoskeleton at physiological insulin concentrations	Balamatsias et al.^90^
**Ras GTPases**				
RalA	vesicles derived from endosomes, GSV	RGC1/2 (GAP)	mobilization of the exocyst complex to facilitate trafficking of GLUT4 vesicles	Chen et al.^91^
		Myo1c	trafficking of GLUT4 vesicles to the cell surface; Myo1c-RalA interaction is modulated by calmodulin	Chen et al.^92^
		Sec5 and Exo84	Sec5 and Exo84 (in the exocyst complex) play a role in vesicle tethering to the cell surface	Chen et al.^93^
		RalGAP	GLUT4 cycling	Skorobogatko et al.^94^
***muscle cells***				
**Rab GTPase**				
Rab7		TBC1D15 (GAP)	TBC1D15 is a master regulator of GLUT4 translocation through late endosomal pathway	Wu et al.^95^
Rab8a	vesicles in perinuclear region	TBC1D1 (GAP), TBC1D4 (GAP), MyoVb	TBC1D4 in myoblasts and TBC1D1 in myotubes are involved in intracellular retention of GLUT4; Rab8A interacts with MyoVb to translocate GLUT4	Ishikura and Klip^96^
		MyoVa	Rab8A-MyoVa mobilizes GLUT4 vesicles toward the plasma membrane	Sun et al.^71^
Rab13	peripheral vesicles	TBC1D4 (GAP)	Rab13 acts at a peripheral step in GLUT4 translocation	Sun et al.^97^
		MICAL-L2	MICAL-L2 links to GLUT4 through filamentous cortical α-actinin-4 enabling their fusion with the membrane	Sun et al.^98^
Rab14	vesicles in perinuclear region	TBC1D1 (GAP), TBC1D4 (GAP)	sorting of GLUT4 from the recycling endosome to the insulin-sensitive compartments	Ishikura et al.^99^
Rab28		TBC1D1 (GAP), TBC1D4 (GAP)	GLUT4 trafficking	Zhou et al.^82^
**Rho GTPases**				
Rac1	cytosol, ruffling area of the dorsal cell membrane		Rac1 stimulates actin cytoskeleton reorganization and activates PAK	JeBailey et al.^100^
			insulin-stimulated glucose uptake is regulated by Rac1 and Akt in parallel pathways; Rac1 involves the actin cytoskeleton reorganization	Sylow et al.^101^
		Elmo2	Elmo2 regulates Akt membrane compartmentalization and Rac1 activation, resulting in enhanced insulin-stimulated GLUT4 translocation	Sun et al.^102^
		Tiam1 (GEF)	AMPK-Tiam1-Rac1 axis mediates contraction stimulated glucose uptake	Yue et al.^103^
		FLJ00068 (GEF)	FLJ00068-mediated Rac1 activation in membrane ruffles mobilizes GLUT4 vesicles	Ueda et al.^104^
			FLJ00068 is a pivotal controller of Akt2-mediated Rac1 activation	Takenaka et al.^105^
		RhoGDIα	RhoGDIα acts as a negative regulator of Rac1 activity and GLUT4 surface transport	M?ller et al.^19^
		PAK1	insulin-promoted GLUT4 translocation	Wang et al.^106^
		PAK1/2	PAK2 is needed, while PAK1 is dispensable for insulin-stimulated glucose absorption in glycotic muscle	Møller et al.^107^
		Arp2/3	Arp2/3 and cofilin coordinate actin cortex remodeling essential for insulin-mediated GLUT4 translocation	Chiu et al.^108^
RhoA			RhoA regulates glucose transport via remodeling of actin cytoskeleton remodeling	Duong and Chun^86^
		ROCK1	GLUT4 translocation and actin cytoskeleton remodeling	Chun et al.^88^
**Ras GTPases**				
		RalA	RalA, regulated downstream of Rac1, exerts a crucial function in GLUT4 surface transport	Nozaki et al.^109^

## 5 Small GTPases and Enzymes of the Mevalonate Pathway in Pathological States of Diabetes and Its Complications

Small GTPases are pivotal in maintaining glucose homeostasis, and aberrant function and regulation of this class of proteins are implicated in the pathological cellular machinery triggered by hyperglycemia. Some reports clearly show glucose-induced upregulation of small GTPases, suggesting that inhibition of such pathways deserves to be considered as a potential therapeutic target in the treatment of T2D and its complications. While expression or activity of Rab members tends to be downregulated under conditions that favor the development of diabetes, overactivated RhoA and Rac1 are involved in many of the pathologies observed in T2D individuals (Table 3). Rac1 is the cytosolic regulatory subunit of the NADPH oxidase (NOX) multicomponent system responsible for ROS generation. Rac1 signaling pathway is implicated in diabetes pathogenesis, mainly by the generation of oxidative stress and islet dysfunction. Hyperactivation of GTP-bound Rac1 is detected in islets derived from T2D patients and animal models.^110^ Importantly, prenylation of Rac1 might be essential for membrane localization and subsequent activation of NOX.^111^ Rac1 activation is also linked to abnormal retinal neovascularization and ROS production, leading to diabetic retinopathy and vascular dysfunction.^112,113^ In the pancreas, hyperglycemic conditions increase RhoA/ROCK activity that contributes to the diminished GSIS^114^ and insulin resistance in muscles.^115^ The progression of diabetic kidney disease^116^ and vascular complications such as diabetic retinopathy or atherosclerosis^117^ have also been connected with elevated levels of RhoA. Taken together, Rac1 and RhoA/ROCK are candidates as new promising targets for pharmacological prevention of islet dysfunction in T2D and T2D-related comorbidities.

**Table 3:** Diabetes-Related Alterations in Ras GTPases and Associated Enzymes of Mevalonate Pathway

GTPase	abnormality	refs
***β-cells***		
**Ras GTPases**		
Rab1a	Rab1a expression is down-regulated in islets of Goto-Kakizaki rats with T2D	Liu et al.^32^
Rab2a	under chronic high glucose, Rab2A effector GAPDH undergoes poly(ADP-ribosyl)ation and dissociation that impairs Rab2A activity	Sugawara et al.^33^
Rab3a	Decreased Rab3a expression under exposure to conditions that promote the development of T2D (proinflammatory cytokines, fatty acids, or oxidized low-density lipoproteins)	Ljubicic et al.^47^
Rab7	Rab7-dependent upregulated RILP expression in diabetic rats or mice causes a reduction of ISGs and promotes proinsulin degradation	Zhou et al.^39^
Rab27a	decreased Rab27a expression upon exposure to conditions mimicking T2D	Abderrahmani et al.^129^
Rab37	decreased Rab37 expression under exposure to conditions that promote the development of T2D (proinflammatory cytokines, fatty acids, or oxidized low-density lipoproteins)	Ljubicic et al.^47^
RhoA	hyperglycemic conditions increase RhoA/ROCK activity that enhances the growth of stress fibers and diminishes GSIS	Kong et al.^114^
RhoA	*RhoA* mRNA levels are higher under lipotoxic conditions in INS cells	Malmgren et al.^130^
Rac1	glucotoxicity results in a sustained hyperactivation of Rac1 targeted to nuclear fraction and induces Rac1-mediated expression of CD36, p53, p38MAPK, and JNK1/2 activation (apoptotic signals, activation of NOX2); Tiam1 and Vav2 contribute to sustained Rac1 activation; prenylation is not essential for nuclear association of active Rac1	Baidwan et al.^110^
	Rac1 prenylation is indispensable for glucose-stimulated NOX2 activation and ROS production	Syed et al.^131^
	Rac1 is translocated to the membrane under hyperglycemia, hyperlipoidemia and increased ROS production	Zhou et al.^132^
	Tiam1 and prenylation-dependent Rac1 activation is pivotal for cytokine-stimulated NOX2 activation and ROS production	Veluthakal et al.^133^
	hyperglycemic conditions increase association between β-PIX (GEF) and Rac1	Damacharla et al.^134^
	Tiam1-Rac1-NOX2 signaling mediates impaired mitochondrial function in the β-cell in response to increased glucose, lipids, or pro-inflammatory cytokines; prenylation of Rac1 is crucial for its membrane translocation and activation of NOX2	Subasinghe et al.^111^
		Syed et al.^135^
	boosts PP2A-Rac1-mediated signaling in metabolic stress-caused β-cell dysfunction	Kowluru^136^
	Rac1- NOX2 signaling pathway induces CD36 trafficking to the cell surface and amplifies influx of free fatty acids resulting in the dysfunction of β-cells	Elumalai et al.^137^
**enzymes of the mevalonate pathway**		
FTase/GGTase-I	high glucose stimulates the expression of the common α-subunit of FTase/GGTase-I without affecting β-subunits and increases the activities of FTase and GGTase-I	Goalstone et al.^126^
	gluco- and lipotoxic ER stress conditions activate caspase-3-mediated cleavage of the α-subunit of FTase and GGTase-I, leading to their inactivation	Veluthakal et al.^127^
***adipocytes***		
**Ras GTPases**		
Rab4a, Rab4b	Rab4a and Rab4b mRNA and protein levels are reduced in epididymal fat in obese diabetic db/db mice; *Rab4b* mRNA expression is decreased in subcutaneous fat in pathologically obese patients with diabetes	Kaddai et al.^138^
Rab5a	*Rab5a* mRNA expression is increased in subcutaneous fat in pathologically obese diabetic patients	Kaddai et al.^138^
Rab11a	*Rab11a* mRNA expression is increased in subcutaneous fat in pathologically obese diabetic patients	Kaddai et al.^138^
Rab18	the presence of Rab18 in human adipose tissue is correlated to obesity; Rab18 overexpression participates in hydrolysis of triacylglycerols	Pulido et al.^139^
	dysregulated production of lumican and GDI2 contributes to IR in obese individuals through modification of collagen I organization and alters lipid storage by inhibiting binding of Rab18 to lipid droplets	Guzmán-Ruiz et al.^20^
RND3	*RND3* mRNA is elevated in obesity and associates positively with insulin resistance; RND3-mediated stimulation of lipolysis leads to insulin resistance; RND3 is farnesylated but it has no intrinsic GTPase activity (insensitive to GAPs)	Dankel et al.^140^
Ras	GGPPS-induced Ras prenylation leads to chronic Erk1/2 signaling in hyperinsulinemia	Shen et al.^15^
**enzymes of the mevalonate pathway**		
GGPPS	Elevated GGPPS expression in insulin-resistant adipose tissues of *ob/ob* mice	Vicent et al.^14^
	hyperinsulinemia stimulates GGPPS and K-Ras by increasing geranylgeranylation; Ras/MAPK/Erk1/2 signaling leads to IRS-1 phosphorylation and insulin resistance; knock-down of *Ggpps* in insulin-resistant adipocytes restores insulin sensitivity	Shen et al.^15^
FTase	hyperinsulinemia promotes the phosphorylation of the α-subunit of FTase and potentiates activation of p21Ras by growth factors	Goalstone et al.^141^
		Goalstone et al.^142^
***skeletal muscle***		
**Ras GTPases**		
Rab1A	Rab1a is upregulated in skeletal muscles of HFD-fed mice and in mitochondria of skeletal muscle from T2D patients	Chae et al.^143^
RND3	defective ROCK1 activity due to increased RND3 expression is connected with insulin resistance in skeletal muscles of obese T2D humans; in mice, ROCK1 deficiency causes whole-body IR as well as defects in insulin signaling in skeletal muscle	Chun et al.^144^
RhoA	RhoA/ROCK signaling under obese and insulin-resistant conditions strains insulin pathway via phosphorylation of IRS-1	Kanda et al.^115^
RhoA	upregulation of mitochondrial RhoA in T2D patients	Chae et al.^143^
Rad	*Rad* mRNA is increased in muscles of T2D individuals; Rad lacks typical prenylation motifs resulting in a primary cytosolic location	Reynet and Kahn^145^
	Rad is increased following insulin stimulation in nonexercised subjects which may be involved in developing insulin resistance in T2D	Coletta et al.^146^
	Rad overexpression inhibits glucose transport in muscle cells	Moyers et al.^147^
	interaction between increased expression of Rad and high-fat diet creates insulin resistance and alters lipid metabolism in T2D	Ilany et al.^148^
**enzymes of the mevalonate pathway**		
GGPPS	GGPPS fosters lipid-induced IR in muscle by activating of the RhoA/ROCK signaling; GGPPS is overexpressed in skeletal muscles of *ob/ob* mice	Vicent et al.^14^
	GGPPS-controlled prenylation mediates lipid-induced insulin resistance by augmenting RhoA/ROCK signaling. ROCK2, but not ROCK1, mediates the GGPPS-regulated PI3K/Akt pathway and glucose transport	Tao et al.^124^
FTase	Reduced insulin-stimulated glucose uptake in muscle is related with augmented FTase expression and more farnesylated proteins	Nakazawa et al.^128^
***liver and nonalcoholic fatty liver disease (NAFLD)***		
Rab24	Rab24 is upregulated in the livers of obese NAFLD patients and positively correlates with increased body fat content. Rab24 inhibition in the liver improves autophagic flux and mitochondrial connectivity, resulting in a reduction in hepatic steatosis	Seitz et al.^149^
GGPPS	GGPPS is highly abundant in mice with obesity and IR	Vicent et al.^14^
	GGPPS is highly expressed in the livers of NAFLD patients; mice with liver-specific GGPPS knockout are protected from HFD-inflicted hepatic steatosis	Liu et al.^150^
	GGPPS deficiency alters the FPP/GGPP ratio; accumulated FPP inhibits *de novo* lipogenesis by activating farnesoid X receptor	Xu et al.^151^
	GGPPS expression is enhanced by lipid overload and regulates hepatocyte-derived extracellular vesicles secretion through Rab27A geranylgeranylation; mice with liver-specific *Ggpps* knockout have a lower fat deposition	Zhao et al.^16^
***diabetic kidney disease (DKD)***		
RhoA	RhoA level is increased in human mesangial cells induced by hyperglycemia and subsequently Rho/ROCK signaling	Chen et al.^152^
	RhoA/ROCK signaling plays a role in the pathogenesis of diabetic kidney disease through glomerular sclerosis signaling pathways and extracellular matrix deposition	Wu et al.^116^
	RhoA translocation to cell membrane is increased in diabetic renal cortex	Massey et al.^153^
***diabetic retinopathy***		
Rac1	activation of Tiam1-Rac1-NOX2 axis in the diabetic retina results in oxidative stress, mitochondrial damage, and cell death.	Kowluru and co-workers^154,155^
	Vav2-Rac1-NOX2 axis is activated in diabetic retinopathy. GDI is decreased in diabetic retinopathy	Mohammad et al.^156^
	Sos1-Rac1-NOX2 axis increases ROS and leads to the pathogenesis of diabetic retinopathy	Mishra et al.^112^
	Rac1 activation is related to impaired retinal neovascularization	Li et al.^157^
	Rac1 activates p38 MAPK and contributes to disruption in the tight junctions, increased vascular permeability and activation of matrix metalloproteinases	Sahajpal et al.^158^
	H-Ras and its effector, Raf-1, are increased in diabetic retinopathy; prenylation of Ras is essential for glucose-mediated effects in the retina in diabetes	Kowluru et al.^159^
FTase	higher FTase levels in retinal microvasculature from humans with diabetic retinopathy; *FNTA* knock-down inhibits glucose-stimulated Rac1-Nox2 signaling	Mohammad et al.^156^
***diabetes-accelerated macrovascular complications***		
RhoA	high glucose increases the growth of VSMCs (vascular smooth muscle cells) and *c-fos* gene expression through RhoA/ROCK	Ishiko et al.^117^
Rac1	high glucose results in membrane translocation of Rac1 leading to NOX activation and ROS generation that promotes proliferation of VSMCs and vascular impairment	Zhu et al.^113^
Ras	high glucose stimulates VSMC proliferation through Ras-Raf-ERK1/2 pathway responsible for atherosclerosis progression	Chen et al.^160^
	hyperglycemic conditions result in Rac1 and endothelial dysfunction with abnormal platelet function.	Schiattarella et al.^161^
HMG-CoA reductase	high glucose induces HMG-CoA reductase overexpression in aortas from diabetics and cultured VSMCs	Chen et al.^5^
FPPS	high glucose induces FPPS overexpression in aortas from diabetics and cultured VSMCs	Chen et al.^7^
GGPPS	high glucose induces GGPPS overexpression in aortas from diabetics and cultured VSMCs	Chen et al.^7^
FTase	high glucose induces FTase overexpression in aortas from diabetics and cultured VSMCs	Chen et al.^7^
	induction of FTase by hyperinsulinemia may account for the proliferative and atherogenic effects of insulin	Draznin^162^
GGTase-I	high glucose induces GGTase-I overexpression in aortas from diabetics and cultured VSMCs	Chen et al.^7^

GTPase can be targeted directly, through their regulatory proteins or prenylating enzymes. This strategy seems to represent a reasonable approach because increased activity of enzymes within the mevalonate pathway was observed in pathological states of insulin resistance, diabetes, and several T2D-related complications (Table 3).

FPPS expression was elevated in cardiomyocytes and aorta cells from diabetic mice with diabetic cardiomyopathy^118^ and atherosclerosis,^119^ respectively. FPPS inhibition by alendronate improved fasting plasma glucose, HbA1c, and insulin resistance,^13^ lowered the high glucose-stimulated proliferation of VSMCs,^7^ and reduced glucose uptake and formation of advanced glycation end products by retinal cells.^120^ Notably, in several clinical trials, treatment with bisphosphonates was correlated with a lower risk of T2D (Table 3). In the context of NAFLD, zoledronic acid attenuated hepatic lipid accumulation and improved liver injury by suppressing RhoA activation via decreasing FPP and GGPP farnesyl diphosphate levels.^121^

GGPPS inhibition may be another therapeutic strategy in T2D settings characterized by GGPPS overexpression. Although GGPPS was reported to decrease in the islets of T2D patients,^122^ this enzyme shows a high expression in the liver, fat and muscles of mice with obesity, IR, and hyperinsulinemia. GGPPS is a crucial mediator linking protein prenylation and metabolic reprogramming, causing NAFLD and subsequent fibrosis development. GGPPS expression was elevated in the livers of mice with obesity-induced hepatic steatosis and NAFLD patients and reduced in hepatocellular carcinoma patients.^123^ In adipocytes, chronic exposure to hyperinsulinism makes GGPPS constantly activated. GGPPS further increased prenylation of K-Ras and induced Erk1/2 activation, IRS phosphorylation, contributing to insulin resistance. Knock-down of *Ggpps* in insulin-resistant adipocytes restored IRS1 phosphorylation and increased insulin sensitivity.^15^ Similarly, in mice fed standard chow and high fat diets, knocking out *Ggpps* in the skeletal muscle increased systemic insulin sensitivity and glucose homeostasis and ameliorated palmitate-induced IR. GGPPS promoted lipid-inflicted IR in skeletal muscles by inducing IRS1 phosphorylation through the geranylgeranylated RhoA/ROCK pathway. Additionally, it was found that ROCK2, and not ROCK1, is involved in the GGPPS-regulated glucose transport in muscle cells, and Rock2 deficiency increases IRS-1/PI3K/Akt signaling in skeletal muscle and insulin sensitivity in the body. Importantly, any changes in muscle properties in the muscle-specific *Ggpps* knockout mice were not observed, suggesting that a deficit of GGPP alone probably does not affect muscle morphology and performance.^124^ Therefore, GGPPS in skeletal muscle and adipose tissue may be a potential pharmacological target for the prophylaxis of insulin resistance and T2D treatment. This method seems to be more selective for GGTase than FPPS targets, as the second approach decreases cellular FPP, which is used in both prenylation and cholesterol synthesis. As a consequence, a GGPPS targeting drug should have a less off-target effect.^125^

Interestingly, short-term exposure of INS 832/13 β-cells and normal rat islets to an insulinotropic concentration of glucose (20 mM) was shown to stimulate the activities of both FTase and GGTase-I along with increased expression of the α-subunit shared between FTase and GGTase-I.^126^ Successively, exposure of INS-1 832/13 cells and normal rodent and human islets to diabetogenic conditions, including long-term exposure to high glucose (30 mM), resulted in a caspase-3-dependent decline in FTase/GGTase-I α-subunit and accumulation of unprenylated Rap1 proteins.^127^ These data provide novel mechanistic insights into regulation of FTase and GGTase activities in the β-cells under normal and glucotoxic conditions. Further studies are required to identify factors regulating the expression and activity of pancreatic prenyltransferases under physiological and diabetic conditions. Especially in insulin-sensitive cells (*e.g.*, muscle, liver, and adipose tissue), significant alterations in FTase and GGTases are connected with insulin resistance (Table 3). For example, in skeletal muscles, increased FTase expression and more farnesylated proteins were linked to decreased insulin-stimulated glucose uptake and metabolic changes. FTase inhibitors induce anti-inflammatory effect preventing inducible nitric oxide synthase (iNOS) expression under pathophysiological conditions.^128^

## 6 Strategies toward Regulation of Activity of Small GTPases via Their Direct Targeting or Inhibition of Mevalonate Pathway Enzymes

The involvement of small GTPases and their prenylation in regulating glucose and lipid homeostasis make this class of proteins important in metabolic disorders.^163^ Here, we summarize the approaches used to regulate GTPase activity that were reported to be associated with T2D. We concentrate on small molecule modulators that have already been used in diabetes-related studies. Simultaneously, we indicate more recent achievements in the field. The stimulus for widening the range of molecular tools comes from the common use of insufficiently potent inhibitors with not fully validated target(s) and selectivity, which might lead to erroneous results.^164^ Therefore, here we highlight the recently introduced compounds of high potency and known selectivity. In many cases, the proposed new molecular tools were applied for cancer-related studies, as small GTPases are commonly dysregulated in malignancies, including pancreatic cancer. We believe that their applicability can be extended to other pathological states.

One of the most typical starting points for studies on the mevalonate pathway and GTPases begins with the observation of the effect of statins on diverse cellular processes. Statins target HMG-CoA reductase, the enzyme at the top of the mevalonate pathway. The question arises as to how the observed effect depends on the more downstream elements of the signaling pathway. It can be further investigated by supplying the system with the missing (due to upstream enzyme inhibition) molecules, geranylgeraniol (GGOH) or farnesol (FOH), or their pyrophosphate analogues GGPP and FPP, respectively. If prenyl alcohols are used, they are converted to the corresponding pyrophosphates in cells and can rescue the effect of the inhibitor. The other solution is to use the inhibitors of more downstream enzymes or compounds interrupting protein–protein interactions to define the genuine target responsible for a particular cellular effect;^165−167^ however, this approach is still under-represented in the literature.

Several strategies can be proposed for the control of small GTPases. First, inhibition of the mevalonate pathway’s enzymes, responsible for supplying the farnesyl or geranylgeranyl pyrophosphates, leads to downregulation of small GTPases. Second, a similar result can be expected from the inhibition of enzymes, which use up these pyrophosphates for prenylation of small GTPases. The third approach involves the interruption of regulatory proteins, such as GEFs, GAPs, and GDIs.^168,169^ Fourth, direct targeting of GTPase, *e.g.*, by modulating oncogenic mutant, K-Ras^G12C^, already resulted in the compound investigated in clinical trials.^170^ Here, we discuss the above strategies and present selected molecular tools that already have been or can be in the future used in studies which aim at deciphering the diabetes–prenylation mutual dependence.

### 6.1 Inhibition of HMG-CoA: Statins

The prenylation of small GTPases requires farnesyl and geranylgeranyl pyrophosphates serving as lipid-donating substrates. These are synthesized via the mevalonate pathway. This route is currently targeted by two classes of drugs, statins, inhibitors of HMG-CoA reductase, and bisphosphonates, inhibitors of FPPS. Their pleiotropic effects are the subject of many studies, aimed at determining the extent to which indirect inhibition of downstream enzymes is responsible for these effects.^165−167^

**Figure 6:**
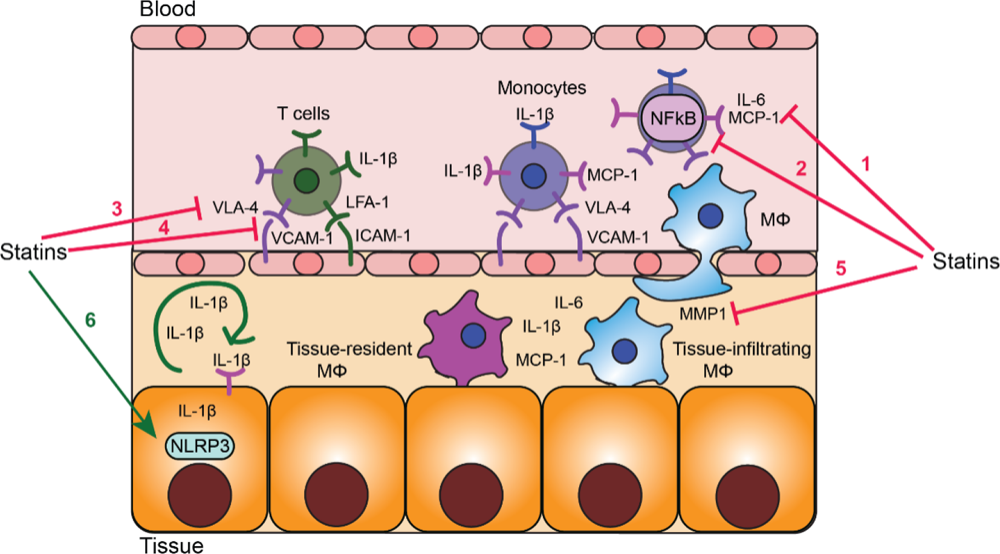
Dual effect of statins on inflammation in diabetes. Statins exert anti-inflammatory effects via (1) reducing chemoattractant levels in the circulation; (2) reducing proinflammatory signaling pathways in blood leukocytes; (3) reducing VLA-4 and FLA-1 integrin levels on blood monocytes and lymphocytes; (4) reducing VCAM-1 and ICAM-1 levels on endothelial cells; (5) reducing MMP1 production by macrophages. These effects result in the inhibition of leukocyte recruitment from the blood into the tissue. Statins exert proinflammatory effects via (6) activation of the NLRP3 inflammasome in insulin-sensitive tissue that leads to enhanced production of IL-1β. IL-1β autostimulation amplifies inflammation and attracts immune cells

Statins are the most prescribed drug regimen for treating cardiovascular disease. Their mechanism of action is based on inhibition of HMG-CoA reductase. However, their structural features differentiate them in terms of potency, solubility, and capability to cross the blood–brain barrier.^166^ Various studies have been devoted to the role of statins in several diseases, besides their original target, cardiovascular disorders. Their effect was observed in cancer, viral diseases, or parasite infections^171,172^ to name just a few. American Diabetes Association 2019 guidelines recommend the use of statins to T2D patients.^173^ Statins have been considered to be anti-inflammatory by inducing the production of anti-inflammatory cytokines which seems to be beneficial for alleviating the systemic inflammation present in diabetic patients. Hyperglycemia promotes inflammation in diabetes by increasing circulating cytokines, activating immune cells, and enhancing their migratory and adhesive capacity. Statin therapy resulted in lower circulating levels of proinflammatory mediators, including C-reactive protein (CRP), IL-1β, IL-6, tumor necrosis factor α (TNF-α), resistin, leptin, visfatin, monocyte chemoattractant protein-1 (MCP-1), intracellular adhesion molecule 1 (ICAM-1), and vascular cell adhesion molecule 1 (VCAM-1), and increased concentration of anti-inflammatory adipokine adopinectin^174−182^ (Figure 6, Table 4). A human pro-monocytic cell line cultured in high glucose and stimulated with LPS showed reduced release of TNF-α, IL-1β, IL-6, and MMP1 after statin treatment.^183^ Inhibition of MMP1 expression by statins was achieved through targeting protein prenylation-mediated ERK activation and could be partially rescued by GGPP. The effect was due to Ras and Rac prenylation as the addition of GGTase-I inhibitor exerted a similar effect to statins.^184^ Moreover, statins lowered resistin expression in 3T3-L1 adipocytes, human preadipocytes and monocytes/macrophages.^175^ Immune cells from diabetic patients who underwent statin therapy showed lower expression of activation markers, lymphocyte function-associated antigen-1 (LFA-1), very late activation antigen-4 (VLA-4), and CD18, and reduced activation potential.^185,186^ Pravastatin and fluvastatin decreased the adherence of neutrophils and monocytes to human endothelial cells under high glucose conditions by reducing the surface expression of endothelial adhesion molecules (intercellular adhesion molecule-1 (ICAM-1), P-selectin, and E-selectin).^187,188^ Furthermore, statin treatment inhibited NF-κBp65 and MAPK proinflammatory signaling pathways in monocytes from T1D patients, muscle cells from streptozotocin (STZ)-treated rats, and aortic endothelial cells cultured under high glucose.^174,189,190^ The effect was H-Ras-mediated, as dominant-negative H-RAs (S17N) exerted an effect similar to that with statin treatment.^190^ Atorvastatin and rosuvastatin improved antigen-specific immunity and cytotoxic activity of T cells in diabetic mice.^191^

**Table 4:**
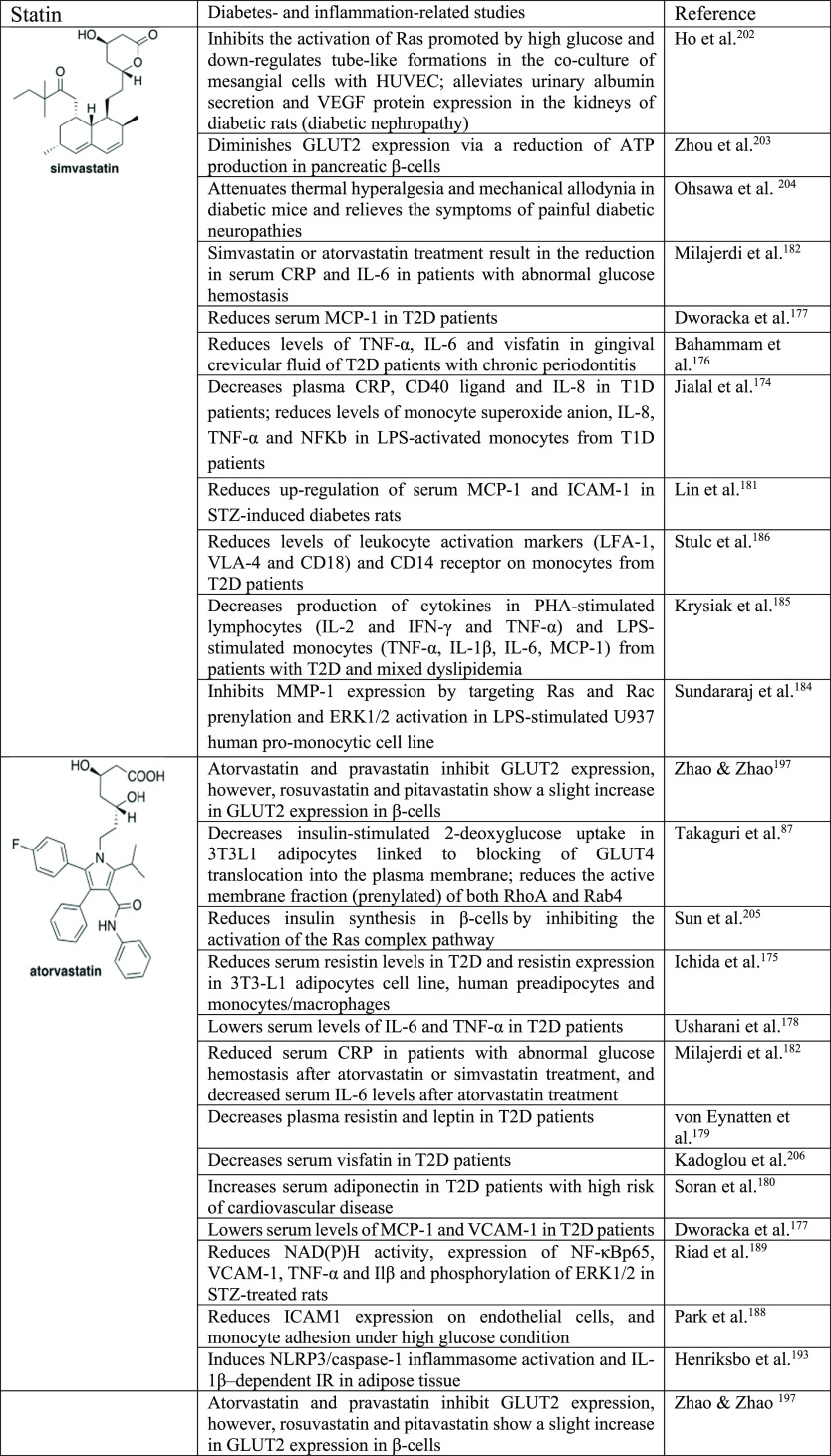

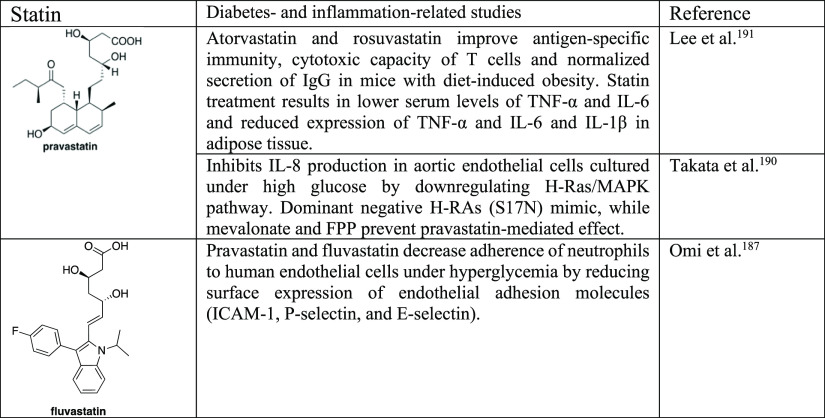
Selected Statins and Their Application as Tools to Study Diabetes and Inflammation^202−205^^a^

a Proinflammatory cytokines: IL-1β, IL-2, IL-6, TNF-α. Proinflammatory chemokines: IL-8, MCP-1. Proinflammatory adipokines: leptin, resistin, visfatin. Anti-inflammatory adipokines: adiponectin. Adhesion molecules: ICAM-1, VCAM-1, E-selectin, P-selectin. Proteases: MMP-1. Signaling pathways: ERK, NF-κB.

However, statins were also demonstrated to contribute to the proinflammatory environments in diabetes. Statins can activate the NLRP3 inflammasome in adipose tissue via p38 and mTOR.^192^ Activation of NLRP3 inflammasome regulates IL-1β, promotes adipose tissue inflammation and leads to IR. The effect of statins was via inhibition of prenylation and not by lowering cholesterol metabolites. The authors studied LPS-primed adipose explants in the presence of either cholesterol derivatives (LDL-cholesterol, free cholesterol or 25-hydroxycholesterol) or GGPP or FOH. They observed rescue in atorvastatin-induced suppression of the insulin signal in fat tissue in the presence of GGPP but not with FOH.^193^

The above studies did not report which of the small GTPases contributed to inflammasome activation and were affected by inhibition of the prenylation. The possible candidates are Rac1, Rap1A, and Rabs. In either statin-treated or GGTase-I-deficient macrophages stimulated with LPS, nonprenylated Rac1 showed increased interaction with its effector proteins, was hyperactivated, and triggered inflammasomes. Preincubating the macrophages with GGPP mostly abrogated the statin effect on cytokine production.^194^ In a statin-treated THP-1 monocytic cell line stimulated with LPS, prenylation of Rabs and Rap1A was inhibited and IL-1β production was induced. The addition of geranylgeraniol (GGOH) restored normal protein prenylation and abolished inflammasome formation and IL-1β and IL-18 release.^195^ In LPS-treated bone marrow-derived macrophages, overexpression of Rab1 increased NLRP3 inflammasomes and IL-1β and IL-18 cytokines, while knockdown of Rab1 or overexpression of its dominant-negative form (Rab1 N124I) had the opposite effect. Whether the effect of Rab1 on inflammasome activation was dependent on its prenylation remains to be assessed.^196^

Overall, treatment of β-cells with statins contributed to a substantial decrease in insulin release. High concentrations of statins induced β-cell apoptosis and further reduced insulin secretion. In addition, by suppressing GLUT4, statins reduce glucose uptake in human skeletal muscle cells and adipocytes.^87,197^ Also, treatment with statins, which results in an increase of cholesterol uptake in the β-cell, leads to reduced protein expression of GLUT2, hence limiting glucose uptake.^197,198^ Inhibition of prenylation using either statins or inhibitors of FTase induced a caspase-3-mediated decline in the levels of prenylated proteins, such as nuclear lamins, leading to β-cell dysregulation and death.^199^ High-dose statin treatment slowed the progression of coronary atherosclerosis, resulting in disease regression in both diabetic and nondiabetic patients.^200^

Although several questions remain unanswered, statins increase T2D risk, with some statins showing a stronger association (*e.g.*, simvastatin, rosuvastatin, and atorvastatin) than others (*e.g.*, pravastatin).^11^ Additionally, as the generation of mevalonate derivatives is blocked by statins and the former regulates the expression of HMG-CoA reductase via multiple feedback mechanisms, there is an observed remarkable increase in HMG-CoA levels. This restricts the effectiveness of the drug and instigates more intensive treatments that may lead to side effects.^201^ Thus, treatment of insulin resistance, T2D, and T2D-related complications with HMG-CoA reductase inhibitors may be a viable option.

### 6.2 Inhibition of FPPS: Bisphosphonates and Nonphosphorus Analogues

The most potent inhibitors of FPPS and GGPPS belong to the bisphosphonates, chemically stable analogues of pyrophosphates, the natural substrates of these enzymes. Bisphosphonate inhibitors of FPPS constitute a known drug class. They bind to hydroxyapatite in bone tissue because of the Ca^2+^ chelating properties of the α,α-bisphosphonic acid motif. They show high selectivity for osteoclasts deposited in bone minerals, and therefore, they are used to restrain osteoclast-mediated bone resorption. Bisphosphonates are also used in patients with cancers causing osteolysis, and some studies show their antitumor activity. However, the charged nature of this group makes them challenging to employ for other therapeutic applications, due to high bone affinity and low serum levels in nonbone applications, low cell membrane permeability, and high clearance by the kidneys. Still, a number of reports have shown that administration of bisphosphonates could be associated with a reduction in the risk of incident T2D,^12^ reduced glucose uptake, formation of glycation end products, insulin resistance,^120^ and hepatic lipid accumulation.^121^ These effects were observed in various tissues affected by diabetes, including the retina and liver (Table 5).

**Table 5:**
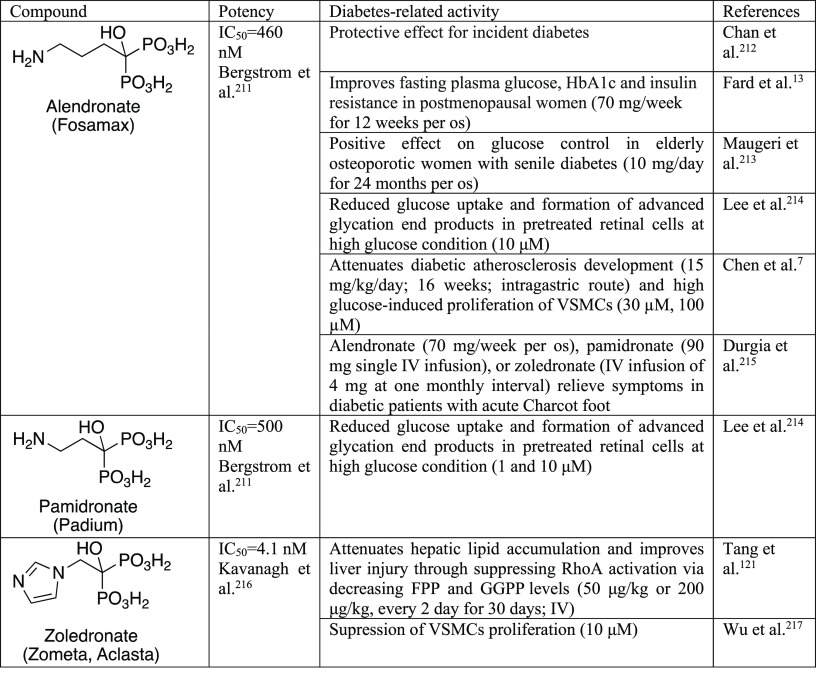
Selected Inhibitors of FPPS^211−217^

Nitrogen-containing bisphosphonates (N-BP), such as zoledronic acid, risedronic acid, alendronic acid, pamidronic acid, and minodronic acid, belong to the clinically validated inhibitors of FPPS (Table 5 and 6). They compete for binding in the allylic site of FPPS with the natural substrates, DMAPP and GPP. The search for inhibitors of human FPPS binding at the active site did not bring nanomolar potency inhibitors without bisphosphonic moiety. Therefore, attempts were directed at identifying inhibitors targeting the allosteric site near the C-terminus of the enzyme.^207^ Several such nonbisphosphonate classes of inhibitors were proposed,^207−210^*e.g.*, **1**–**4**, although not all of them bind inside the FPPS allosteric pocket.^210^ Although these compounds were designed to have superior “druglike” properties in comparison to the bisphosphonates, none of them showed notable antitumor activity in cell-based tests. To the best of our knowledge, their potential in diabetes-related studies has not been investigated yet. That is why here we show only selected examples, limiting cases to those tested for human FPPS and showing nanomolar potency (Table 6).

**Table 6:**
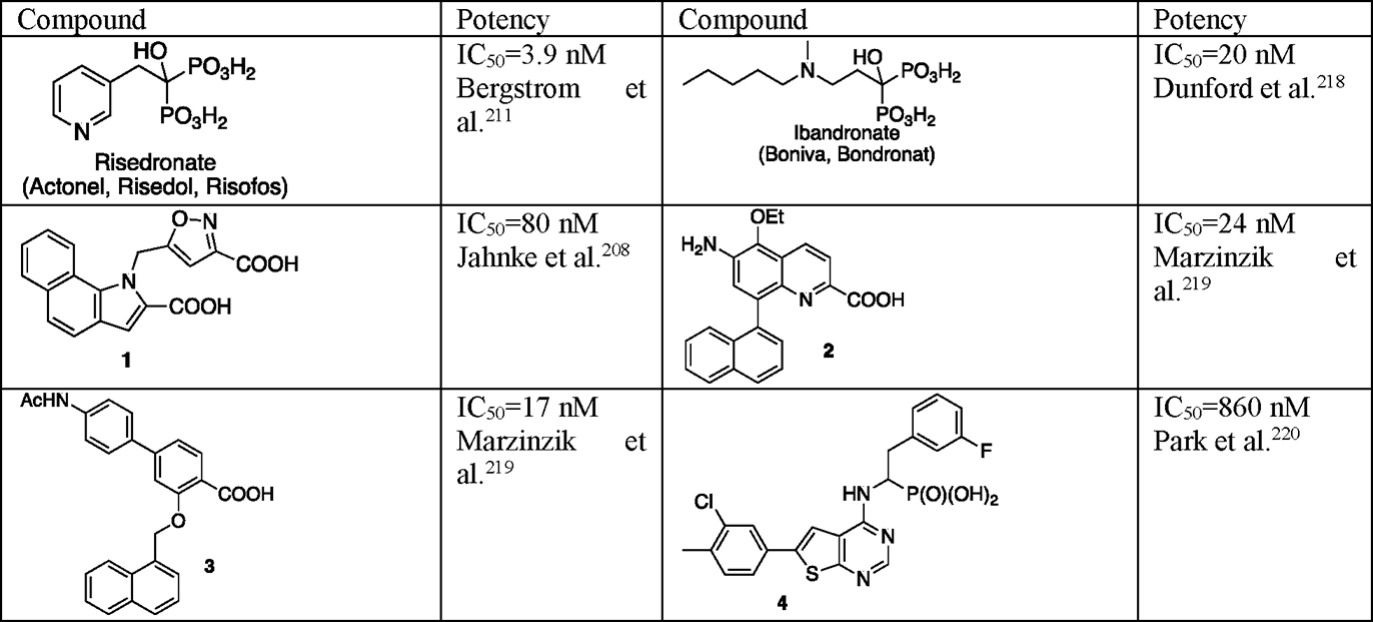
Bisphosphonate and Non-bisphosphonate Inhibitors of FPPS with Potential to Be Used in Diabetes-Related Studies^218−220^

### 6.3 Inhibition of GGPPS: Lipophilic Bisphosphonates

The enzyme responsible for the synthesis of geranylgeranyl pyrophosphate is GGPPS, and it is now intensively studied as a potential drug target.^221^

The elevated expression of GGPPS was induced by high glucose levels.^7^ Its high abundance was observed in a number of tissues of obese and/or diabetic patients, promoting, for example, lipid-induced muscle insulin resistance.^14^ However, up to now, the GGPPS inhibitors were not used in diabetes-related studies. Instead, inhibitors of upstream enzymes in the mevalonate pathway were applied or the experiments were run on cells with *GGPPS* knock-down. Therefore, here we show that direct inhibitors of GGPPS do exist and we present the selective and the most potent among them as available chemical tools to study diabetes-related processes.

**Figure 7:**
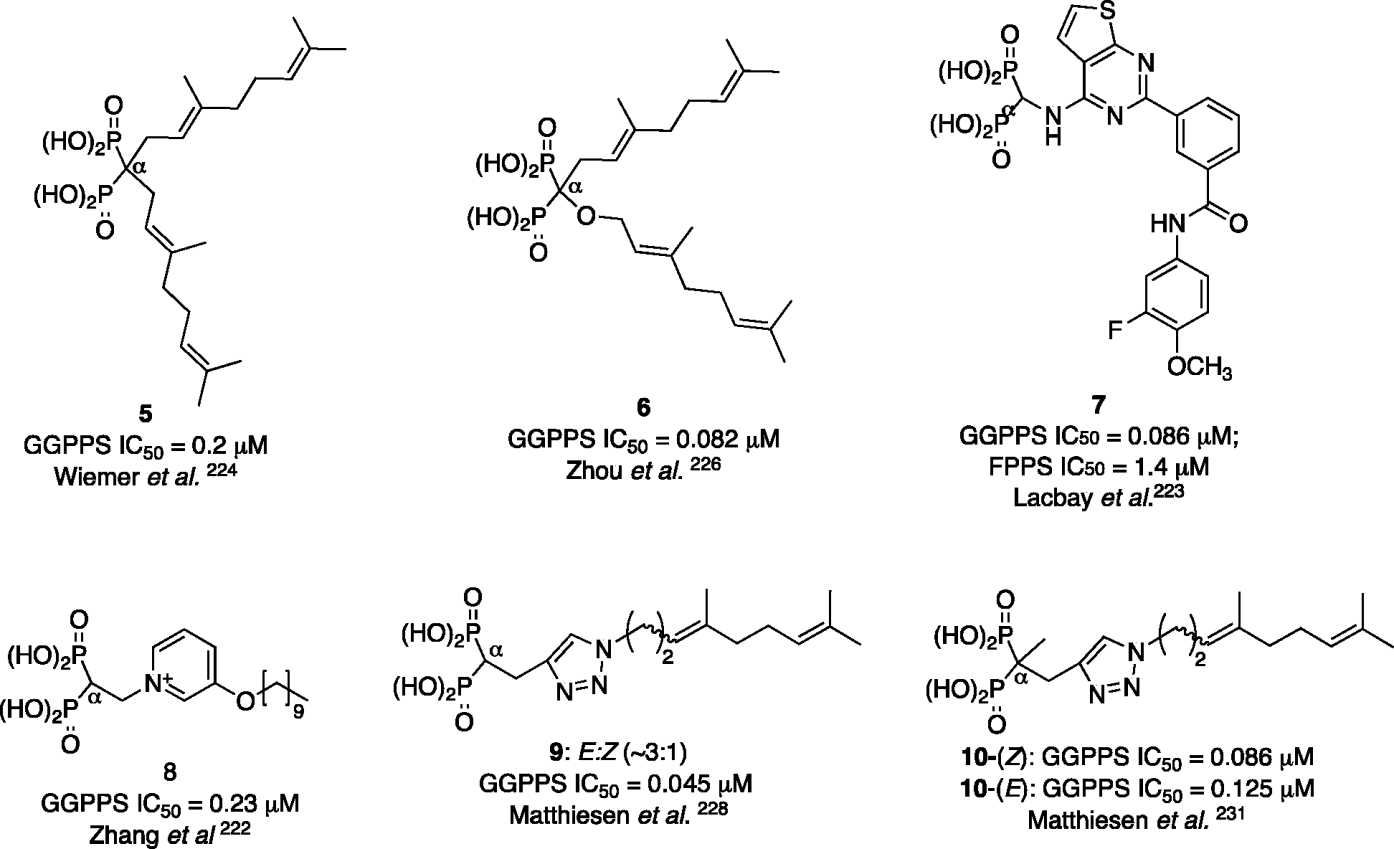
Structures of the selected GGPPS inhibitors not used in diabetes studies.

The number of selective GGPPS inhibitors is limited, partially due to the previously held conviction that dual FPPS and GGPPS inhibitors are more efficient as antitumor agents. Despite the low sequence identity between human FFPS and GGPPS (17%), their tertiary (but not quaternary) structures are surprisingly similar and their catalytic mechanisms are probably similar.^207^ Therefore, many attempts at obtaining GGPPS inhibitors led to the development of dual FPPS and GGPPS inhibitors, such as compound **8** (Figure 7), which is about 100 times more potent than zoledronic acid in obstructing tumor growth,^222^ or compound **7**, which represents another chemotype of GGPPS bisphosphonate inhibitors and shows ∼15× higher activity toward GGPPS, compared with FPPS.^223^

The FPPS inhibitors are usually smaller molecules, having a shorter alkyl chain and a positive-charge feature. The GGPPS bisphosphonate inhibitors contain one or two large hydrophobic groups, they lack hydroxyl group in C-α, and there is no positive charge required. Therefore, they are more lipophilic, which makes them more prone to targeting nonbone tissues.^207^

The broadest class of GGPPS inhibitors contains a bisphosphonic acid moiety, which is a substitute of the unstable pyrophosphate residue. It turned out that digeranylated bisphosphonic acid **5**, representing the so-called V-shaped molecules, shows 0.2 μM activity against GGPPS and no inhibition of farnesylation.^221,224^ At least one geranyl or longer isoprenoid chain is required for inhibition of GGPPS; these prenyl chains occupy the substrate and product binding sites, FPP and GGPP, respectively.^225^ Several such V-shaped compounds,^224,226^ including those that contain an ether bond, **6**,^226^ and the so-called U-shaped analogues were prepared.^227^

Recent works show the anticancer therapeutic potential of several hydrophobic bisphosphonates. However, the most interesting group is constituted by triazoles^228^ that carry an isoprenoid chain (Figure 7). The homogeranyl and homoneryl triazole analogues, **9**, turned out to be the most potent GGPPS inhibitors reported, demonstrating high selectivity in inhibiting GGPPS vs FPPS. They can slow pancreatic tumor growth *in vivo*.^229^ The preliminary studies on metabolic stability and pharmacokinetics indicate that they are metabolically stable in human liver microsomes.^230^ Most analogues showed a higher potency of the *Z* isomer. An interesting property was observed for **9**, as studies demonstrated that the two isomers interact synergistically, making the mixture more potent than a single isomer. It is tentatively explained as resulting from synergistic binding in both the substrate, FPP, and product, GGPP, inhibitory channels.^221^ In the case of analogues bearing a methyl group at C-α, compound **10**, the activity against GGPPS was similar for both isomers, 0.086 mM for (*Z*)-**10** and 0.125 mM for (*E*)-**10**.^231^ Additionally, such a design, with the locked C-α, enables the prodrug form preparation to overcome the bioavailability hurdles of bisphosphonic drugs.^231^

### 6.4 Inhibition of Prenylating Enzyme, FTase, and Direct Targeting of Ras Proteins

Ras proteins regulate cell proliferation, differentiation and survival. The most known members of the Ras subfamily are Harvey-Ras (H-Ras), neuroblastoma-Ras (N-Ras), and Kirsten-Ras (K-Ras). K-Ras is the most commonly mutated protein in many cancers, accounting for almost 85% of all Ras mutations.^232^ The K-Ras^G12D^ mutation is the most prevalent in pancreatic and colorectal cancers. G12 is located at the protein active site, interacting with a phosphate-binding loop (P-loop) and two switch regions, which control binding to effector and regulatory proteins. The oncogenic K-Ras mutation inhibits GTP hydrolysis (by weakening its GTPase activity or hampering the GAP-stimulated GTP hydrolysis), making such mutants constantly active and activating downstream effectors.^233^

In the early efforts to control the activity of Ras, the inhibition of FTase was the most widely developed approach. FTase is responsible for PTMs of Ras, enabling their proper localization in the membrane, often after additional modifications, such as palmitoylation. While several FTIs (FTase inhibitors) were developed, they failed in clinical trials due to alternative prenylation with GGTase-I, which restored their membrane association. There is renewed interest in FTase inhibitors, as their efficacy against the regulation of H-Ras activity has been verified. Out of a few dozen trials, one FTI small molecule drug, lonafarnib (commercially available from Sigma-Aldrich), has been recently approved by the U.S. Food and Drugh Administration [FDA; https://www.fda.gov/drugs/drug-approvals-and-databases/drug-trials-snapshots-zokinvy] for the therapy of Hutchinson-Gilford Progeria Syndrome and certain progeroid laminopathies. Several other drug candidates are at various stages of preclinical or clinical trials to prevent or treat cancer, such as manumycin-A, FTI-277, tipifarnib, L778123, and BMS-214662.^170^

Several other strategies directly targeting Ras proteins have been developed. Besides the use of biologics, such as monoclonal antibodies, mimetics of antibody variable fragments, and antisense oligonucleotides,^234^ efforts have been undertaken to interrupt the association between Ras and regulatory or effector proteins, such as phosphodiesterase-δ, Sos, Raf, or Tiam1. A breakthrough strategy has been developed for selective targeting of a mutant variant of K-Ras^G12C^ and small molecules, such as AMG510, MRTX849, ARS3248, and LY3499446 covalently modifying the mutant cysteine, that has progressed to clinical trials (*e.g.*, NCT04380753, NCT04667234).^235^ Recently, Crews and collaborators have shown the potential of a PROTAC molecule, LC-2, developed from the covalent K-Ras^G12C^ inhibitor (MRTX849) linked with the VHL (von Hippel-Lindau ligase) ligand, which turned out to be an efficient K-Ras degrader.^236^ Several reviews have been recently published covering these topics [see refs (232) and (235)].

Few studies were devoted to selective targeting of another mutant K-Ras^G12D^, the most prevalent in pancreatic cancer. Sakamoto et al. introduced K-Ras^G12D^ KS-58, derived from KRpep-2d (Ac-RRRRCPLYISYDPVCRRRR-NH2), which inhibited interactions with two proteins, RasGDP-Sos1 (GDP-GTP exchange) and RasGDP-BRaf. It inhibits both GDP- and GTP-bound K-Ras^G12D^. Despite its molecular weight (1333.6 g/mol) and negatively charged polar residue, it showed anticancer activity *in vivo*, making it a potential lead compound.^234^

To the best of our knowledge, Ras proteins have not been directly associated with diabetes yet, as their misregulation is more connected with cancer. However, several reports indicate that hyperglycemia and/or hyperinsulinemia stimulate the expression and/or activation of FTase (Table 3). Therefore, we listed some FTase inhibitors (Table 7), concentrating on those that have been already used in diabetes-related studies or are at various stages in clinical trials. Most of them are commercially available, which makes them accessible for many laboratories. On the other hand, the repurposing strategy for already studied (potential) therapeutics has many advantages. Such agents have already undergone thorough examinations in terms of their toxicity, bioavailability, and other aspects, which need consideration in drug development. For more information on the plethora of FTase inhibitors, please refer to recent reviews [see refs (232) and (235)].

**Table 7:**
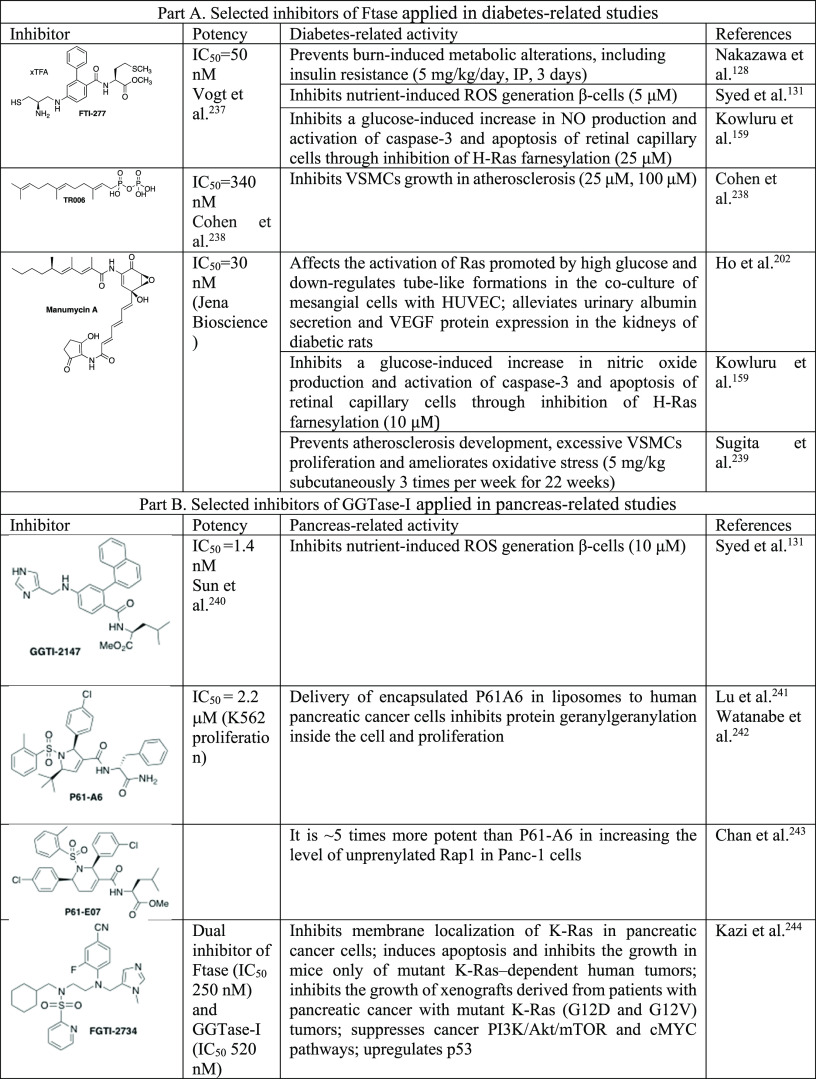
Selected Inhibitors of FTase, GGTase-I, and Ras Proteins^237−240,243^

### 6.5 Inhibition of Prenylating Enzymes: GGTase-I

GGTase-I inhibitors have received less attention than inhibitors of FTase. GGT-I inhibitors often serve in combination with FTIs in order to inhibit prenylation and function of oncogenesis drivers, K-Ras and N-Ras proteins. Blocking only FTase activity led to alternative prenylation of FTase substrates by GGTase-I. Therefore, several dual inhibitors of these two prenyl transferases were also developed.^244^

Interestingly, this research area also evolved in a different direction: the development of agents directly targeting the GGTase-I substrates, Rho GTPases. This gives an alternative pathway for the selective regulation of particular GTPases. This topic is covered in the following paragraph.

Although GGTase-I is an attractive target for cancer-related studies, its inhibitors are rarely used in diabetes research. GGTase-I might be overexpressed under high glucose concentrations (Table 3), while its knock-down blocked diabetes-accelerated atherosclerosis,^251^ which might be related to interfering with Rac1 geranylgeranylation, finally inhibiting ROS production, and ERK1/2 and JNK signaling.

Peptidomimetics of the CAAX motif in protein substrate and dihydropyrrole or tetrahydropyridine-based analogues constitute two main classes of GGTase-I inhibitors. Here, we listed inhibitors of GGTase-I, giving priority to molecules that have already been used in diabetes-related studies. Among them, we find selective a GGTase-I inhibitor, GGTI-2147, and FGTI-2734, which show dual inhibition of FTase and GGTase-I.^244^ The representative of dihydropyrrole analogues, P61-A6,^242^ was applied in the design of targeted delivery of P61-A6 to pancreatic cancer cells.^241^ For that purpose, the GGTase-I inhibitor (or in combination with FTase inhibitor) was encapsulated into liposomes, which upon exposure to the lower pH of cancerous cells was released.

There are some representatives of GGT-I inhibitors, which have potential in future studies as they are of nanomolar potency, are commercially available and commonly applied in biological studies, or show different degrees of selectivity against FTase vs GGTase-I. We also include GGTI-2418 as the only GGTase-I inhibitor currently in clinical trials. Selected examples of such compounds are listed in the Tables 7 and 8.

**Table 8:**
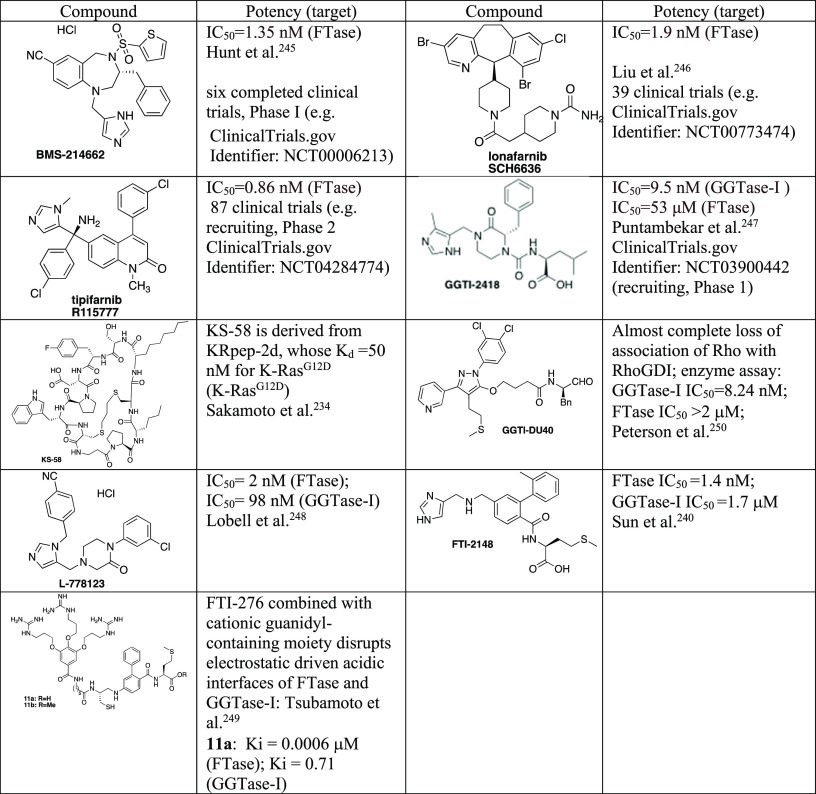
Selective and Dual Inhibitors of FTase and GGTase-I and Direct Inhibitor of K-Ras that Have Potential to Be Used in Diabetes-Related Studies^245−250^

### 6.6 Direct Targeting of Rho GTPases

The strategy based on inhibition of GGTase-I alone or in combination with FTase is limited by its nonselectivity in terms of affecting many GTPases. The efforts to directly and selectively target Rho GTPase ended with success. The most studied representatives of Rho GTPases are Rac1, RhoA, and Cdc42, which are often overexpressed in malignancies, as they are regulators of cancer cell migration and invasion. The subfamilies of Rho GTPases interact with each other and are controlled by regulatory proteins and effectors.^252^ Their hyperactivation can result from their mutations, downregulation of GAPs, or upregulation of GEFs. The latter interaction is the most commonly targeted. As the topic of regulation of Rho GTPases has been widely summarized recently,^253,252^ here we concentrate on selected inhibitors, directly targeting Rac1 and RhoA, as the connections of these with diabetes-related malfunctions are the most broadly reported (Table 9).

**Table 9:**
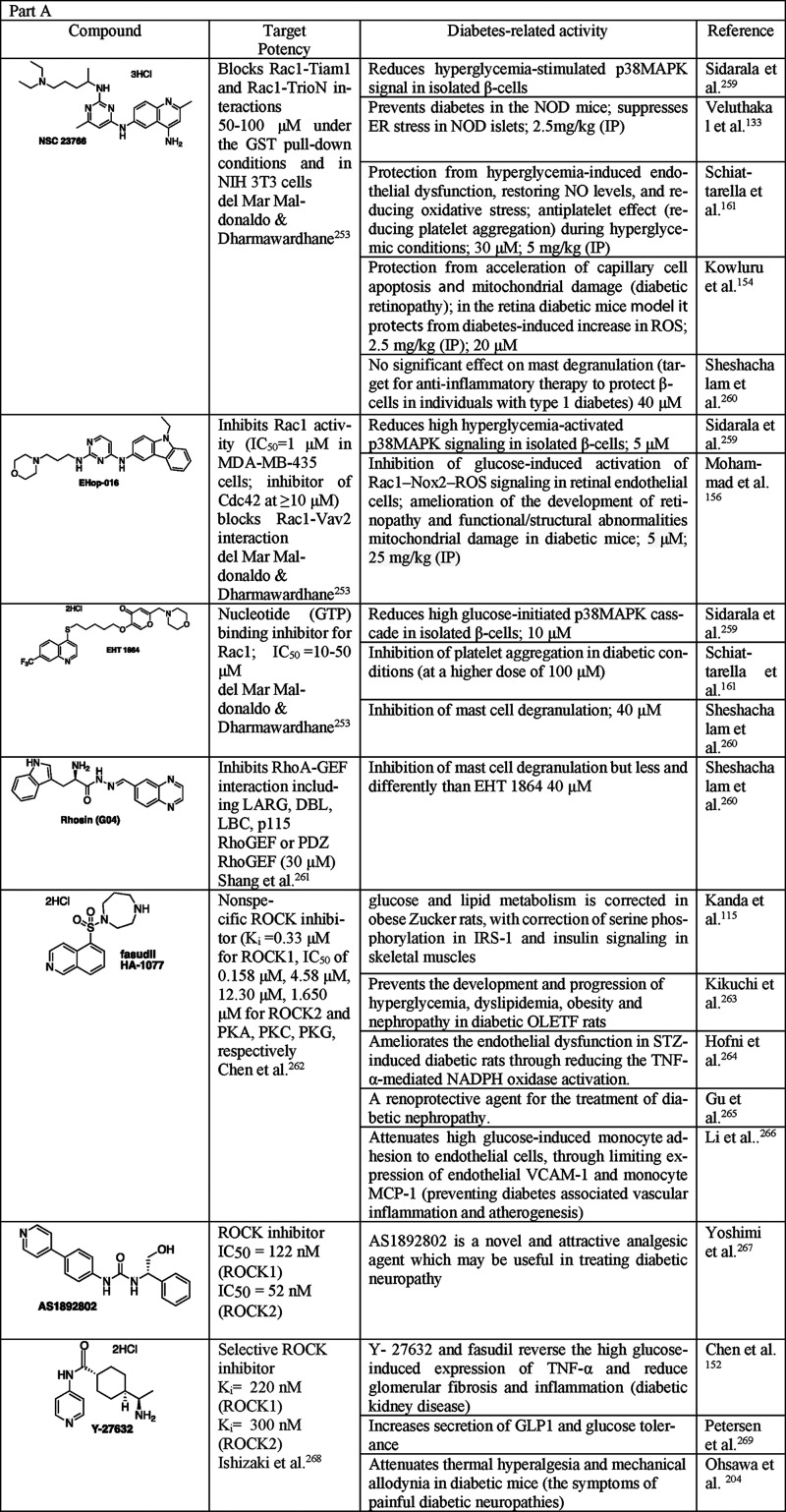

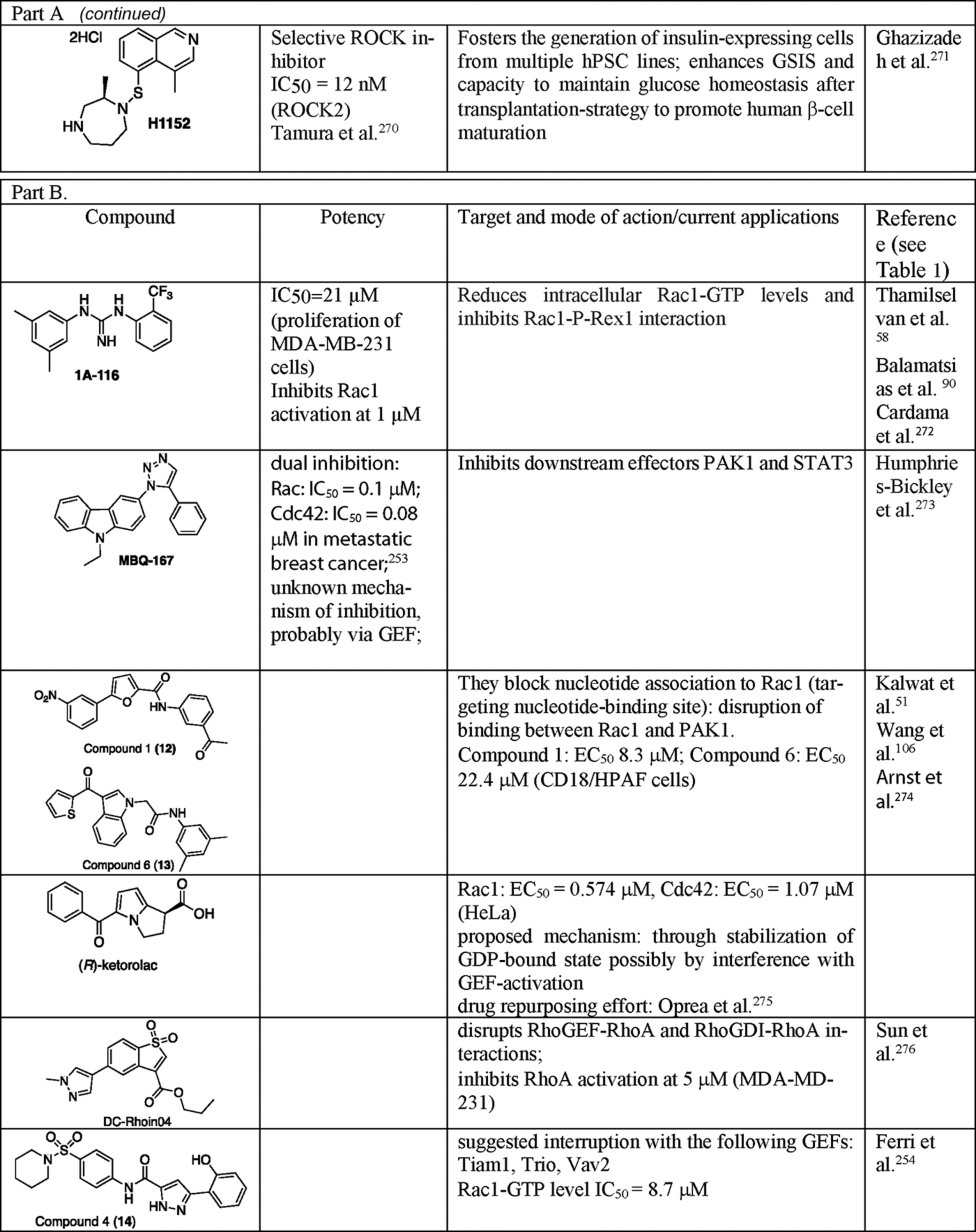
Compounds Interrupting the Protein–Protein Interactions of Rho GTPases Applied in Diabetes-Related Research (Part A) and Those That Have Potential to Be Used in Future Diabetes-Related Studies (Part B)^259−276^

As has been already mentioned, one of the most popular strategies to inhibit Rac1 activation is the interruption of its binding with GEFs. There are several Rac1-Tiam1 (GEF) (T-cell lymphoma invasion and metastasis 1) inhibitors.^254−257^ The structural studies identified the specific amino acid residues.^253^ In addition to small molecule inhibitors, there were attempts to develop peptide-derived Rac1-Tiam1 inhibitors.^258^

In the case of RhoA regulation, it was found that GGPPS promotes lipid-induced insulin resistance in muscle by enhancing RhoA/ROCK signaling.^124^ It could be prevented by inhibition of GGPPS or RhoA/ROCK interaction. Several ROCK kinase inhibitors have been developed and used as tools in diabetes-related studies (Table 9). However, one needs to remember that the ROCK pathway is essential for many cellular processes and Rac and Cdc42 are crucial regulators of a plethora of cell signaling receptors.^253^ Therefore, more selective approached are needed.

In Table 9, we present inhibitors that can potentially be used as probes, as they interrupt protein–protein interactions that are important in diabetes. Among them, we can distinguish inhibitors of Rac1 interaction with GEFs such as P-Rex1, Vav2, or Trio. Another mechanism works for compound **12** and **13** that by blocking interaction with nucleotide disrupts binding between Rac1 and PAK1.

### 6.7 Inhibition of Prenylating Enzymes: GGTase-II

The abnormal activities of GGTase-II and some Rab proteins have been identified in several diseases, including cancer, such as pancreas, breast, skin, colon, lung, ovarian, and prostate, to name just a few.^277^ GGTase-II alone was not reported to be up- or downregulated in diabetes, but some Rab GTPases can be associated with various aspects of T2D (Table 3). Up to now, in most identified cases, the pathological effect of dysregulation of Rab GTPases was associated with their impaired activity. However, in a few cases, Rab GTPase was upregulated, *e.g.*, Rab24 in the livers of obese NAFLD patients correlated with body fat content.^149^ Since the current state of knowledge implies that, in diabetes, the upregulation of Rabs is required to reverse the pathological state, new strategies need to be developed. Here, we discuss the approaches that have been studied to date to present the currently available tools.

**Figure 8:**
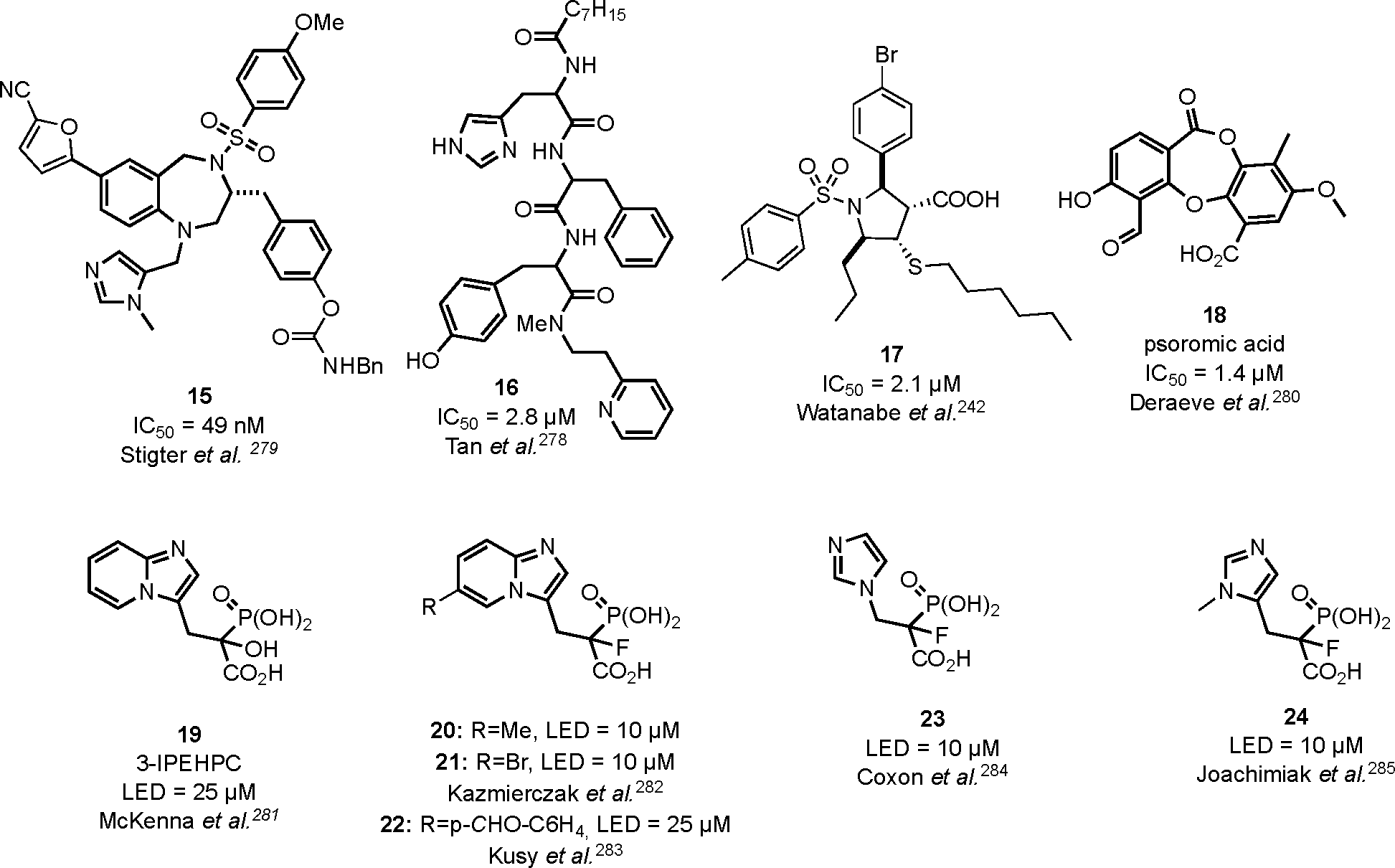
Structures of the selected GGTase-II (RGGT) inhibitors not used in diabetes studies. LED: lowest effective dose toward inhibition of Rab11 prenylation.

Several attempts have been made to control GTPases; however, these approaches are not very diversified. One of the most studied strategies is based on the development of inhibitors of GGTase-II. This enzyme was proven to be a druggable target. Several classes of small molecule inhibitors have been developed (compounds representing these classes (**15**–**24)** are presented in Figure 8),^242,278−281^ differing in their mode of action (*e.g.*, inhibitors of first or second geranylgeranylation), selectivity (versus other prenyltransferases), and potency. GGTase-II inhibition is limited by the lack of substrate selectivity, as it affects all or most Rab GTPases. The most active analogues contain a tetrahydrobenzodiazepine motif (compound **15**).^279^ Only in the case of α-phosphonocarboxylates (**19**–**23**), the selectivity toward different Rabs was reported. This class of inhibitors prohibits the introduction only of the second geranylgeranyl group to Rabs, leaving the monogeranylated Rabs unaffected. Among the currently known phosphonocarboxylates, the most active ones contain imidazo[1,2-*a*]^282,283,281^ or the imidazole ring.^284,285^

Another strategy is based on the direct targeting of Rab GTPases. Only few such attempts have been reported in the literature. These studies involved analysis of the protein–protein interaction surfaces in order to design molecules mimicking them. These studies resulted in the development of stapled peptides, StRIP16, which targets Rab8a, mimicking its interaction with RIP,^286^ and RFP14, blocking Rab25:FIP complex formation, in which FIP is the effector protein.^287^ Although these studies were also dedicated to optimizing the stability and bioavailability of these inhibitors, they need further refinement.

## 7 Recent Strategies for Selective Targeting of Inhibitors to Diabetes-Affected Organs

The small GTPases and their regulatory proteins are omnipresent in all kinds of cells. Therefore, when planning to use the inhibitors in diabetes-related studies, specific delivery to certain tissues needs to be considered to increase their efficiency and bioavailability while reducing toxicity and dosing frequency. A number of reviews exist that describe organ-specific delivery systems^288^ and prodrug strategies, including those that show a possible masking of ionic phosphonic groups, with the latter being so popular among the compounds described in this Perspective.^289^ Here we selected several approaches targeting tissues related with diabetes.

The development of various types of antidiabetic drugs has been accompanied by the constant progress in the field of their delivery, especially in terms of the effective and convenient transport of insulin, a protein, which due to its unstable nature cannot be delivered orally. Peptide-derived therapeutics have limited oral bioavailability due to their destruction by gastric acid and proteolytic enzymes and the limited absorption from the intestine. However, medicinal chemistry has developed several strategies to overcome these hurdles, based on various structural modifications (*e.g.*, PEGylation, attachment of cell-penetrating peptides) or coapplication of enzyme inhibitors. That topic has been broadly described in many medicinal chemistry textbooks. In the case of peptides and other classes of therapeutics, the transportation and targeting can be improved by the use of nanocarrier delivery systems, which include liposomes, niosomes, polymeric nanoparticles or micelles, and dendrimers.^290^ When the drug is encapsulated within a nanostructure, such a nanomaterial presents both opportunities, such as the possibility of surface modification with a tissue-targeting moiety as well as safety concerns, variable efficiency, outcome of biomaterial degradation, and possible side effects. The field of nanodelivery is under constant development, and one needs to be aware that such studies require additional caution, but the potential of nanocarriers cannot be denied. Here, we present examples of the recently reported strategies or reviews for selectively targeting drugs to β-cells, liver cells, adipocytes, and muscle cells.

The interesting feature of β-cells is an exceptionally high concentration of zinc ions (up to ∼30 mM) while the zinc concentration in the cytosol in most cells is ∼400 pM.^291^ Zn(II) can catalyze hydrolytic reactions, which can be used to ignite the activity of the released cargo. Because of the above features, many attempts were reported to design a system for imaging β-cells.^292^

That feature was used for attaching a zinc-chelating residue onto a β-cell replication-inducing compound.^293^ Another study involved designing a prodrug consisting of an inactivated drug linked with a Zn(II)-binding ligand. Such an approach was applied for the targeted release of fluorochromes and β-cell mitogenic compounds in human β-cells.^292^ In both cases, the hybrid compounds preferentially accumulated within β-cells. Upon reaching the Zn(II)-abundant environment, the bond between the cargo and the Zn(II)-binding scaffold was cleaved, releasing the active cargo.

In the last 20 years, diverse strategies have been developed for noninvasive imaging of β-cells for diagnostics. For that purpose, a number of β-cell-surface-specific proteins, often overexpressed, were used, such as vesicular monoamine transporter 2 (VMAT2), sulphonylurea receptor (SUR-1), glucagon-like peptide 1 (GLP-1), free fatty acid receptor 1 (FFAR1), and β-cell-specific antigens. Some of the markers used for β-cell imaging can be used to design targeting molecules, such as monoclonal antibodies, to selectively deliver a drug, which will be cleaved upon reaching the target.^294^ To recognize the surface-specific protein, antibody–drug conjugates could be used, which recently have gained importance as an attractive approach for cell-specific targeting. Although challenging, GPCR-specific monoclonal antibodies are also being developed, and the first ones, erenumab and mogamulizumab, were recently approved by the FDA.^295^

These strategies were developed for certain tissues affected by nondiabetes-related pathological states, such as cancer, liver fibrosis, and muscle aging. Analogous strategies can be applied for the targeted delivery of drugs to the tissues affected by diabetes. Still, careful evaluation needs to be conducted to determine to what extent the developed methods can be applied for diabetes-stricken organs.

For selective targeting to the liver, several delivery methods, including the ones that use surface markers, were developed for liver cancer cells^296^ and proposed for liver fibrosis.^297^ In the case of muscle cells and adipocytes, selective targeting is challenging because of their high representation in the body. However, for skeletal muscle, surface recognition elements were identified and used for selective uptake. In addition to small molecules like carnitine (a drug linked with carnitine shows improved muscle uptake via OCTN2 transport), monoclonal antibodies, or viral vectors,^298^ aptamers have also been proposed as a muscle-specific delivery vehicle.^299^

## 8 Future Perspective

The involvement of small GTPases and their prenylation in regulating glucose and lipid homeostasis makes this class of proteins important in metabolic disorders. Inhibitors of protein prenylation have been investigated as potential therapeutics to treat multiple diseases. Statins, used primarily as cholesterol-lowering drugs, were also found to reduce systemic inflammatory responses independently of cholesterol. Various clinical trials demonstrated that treatment with statins decreased soluble proinflammatory mediators and lowered the activation capacity of monocytes and lymphocytes.^176,177,179,182,206^*In vitro* studies identified statin targets as being small GTPases (Ras, Rac and Rho).^174,184,190^ On the other hand, accumulating evidence suggests that statins enhance the inflammatory responses and elevate the risk of diabetes.^11^ The evidence for statin-mediated effects points toward the NLRP3 inflammasome/caspase-1 complex, and this could be a new target in the treatment of inflammation in diabetes.^192,193^ However, there may be more still-unexplored prenylation targets that contribute to increased inflammation upon exposure to statins. Thus, decreasing the activity of enzymes that are downstream from HMG-CoA reductase in the mevalonate pathway may be a promising strategy for treating insulin resistance and diabetes. Pro- and anti-inflammatory effects of statins could be explained by the opposite outcomes of the mevalonate pathway’s inhibition, depending on the tissue, euglycemia versus hyperglycemia, and target type. Enhancing prenylation may localize specific GTPase and thus enhance its function. It may also sequester it away from its effectors and reduce the effect. Further studies should be conducted to assess how prenylation controls inflammation and insulin sensitivity in muscle, liver, and adipose tissue, and insulin production and secretion by pancreatic islets. Statins, inhibitors of other enzymes in the mevalonate pathway, as well as GTPase activation inhibitors should be employed to identify the specific factors that enhance or reduce inflammation and contribute to insulin resistant β-cell dysfunction. It will further our knowledge about the function of prenylation in diabetes and allow the development of more context-specific treatments.

Defective or upregulated prenylation can contribute to the decrease of metabolic cell viability and dysfunction in pancreatic β-cells.^127^ Several enzymes are decreased in the islets of T2D patients while they are upregulated in the liver, adipose tissue, and muscles in individuals with obesity, insulin resistance, and hyperinsulinemia (Table 3). Therefore, further studies are required to identify factors regulating the expression and activity of pancreatic prenyltransferases under physiological and diabetic conditions. More work needs to be done to show which signaling pathway is essential for desired efficacy. Moreover, a better understanding of how the beneficial effect from preclinical T2D models can be effectively translated to T2D patients is needed.

After a broad search for the interconnections between small GTPases and different proteins and processes in T2D, we summarized the approaches that can be used to regulate GTPases activity in pathological cellular machinery triggered by hyperglycemia. We concentrated on small molecules. It is crucial to be cautious when using inhibitors, both those newly reported as well as such that are known for some time. The proper molecular probe should be potent and selective toward the validated molecular target. Otherwise, such studies might repeatedly generate uncertain or even erroneous results.^164^ Therefore, here, besides showing the previously used chemical probes, sometimes not of the highest quality,^164^ we highlight the recently introduced compounds of high potency and known selectivity.

We described the most common strategies used to control small GTPases, via inhibition of the mevalonate pathway and prenylating enzymes, or the interactions between GTPases and their regulatory proteins, such as GEFs. In the case of most GTPases, there has been significant progress in developing chemical tools—potent and selective inhibitors—allowing further studies. However, most approaches studied involve the downregulation of GTPases, while expression or activity of Rab GTPases tends to be downregulated under conditions that favor the development of diabetes. In addition to targeting the gene expression, no other strategy to achieve Rab upregulation has been applied yet. Here, the opportunity might be spotted at targeting the interactions with regulatory proteins, such as GAP and GDI, which bind Rabs and inactivates them under normal circumstances. Also, downstream effectors, or other post-translational modifications, such as phosphorylation/dephosphorylation, ubiquitination, palmitoylation, and serotonylation, can be targeted.^253,300^

In diabetes-related studies, the apparent targets among GAPs constitute TBC1D1 and TBC1D4, which are Akt targets in insulin-stimulated GLUT4 traffic. Mutations in TBC1D1 and TBC1D4 are linked with obesity and insulin resistance in humans. Phosphorylation of TBC1D1 and TBC1D4 is thought to shut down their GAP function, leading to increased levels of active Rab GTPases, which triggers GLUT4 translocation.^301^

However, these different approaches are not straightforward. Individual functions of the different Rab proteins that undergo various post-translational modifications, such as phosphorylation, serotonylation, AMPylation, phosphocholination, palmitoylation, and ubiquitination, often occur at localization, which affects the interaction with diverse proteins GAPs, GDIs, and effectors. Only a few such interactions have been already identified, and only in a few cases it was determined when the interaction with the effector is taking place, after or before particular post-translational modification. Phosphorylation of Rabs is still poorly recognized in terms of its role, mechanistic implications, and regulation via kinase-phosphatase-mediated modifications. The different sites might be phosphorylated by different kinases, leading to diverse effects and distinct distribution of Rabs, altering the activity of GAPs, GEFs, effectors, and others. Also, phosphorylation of Rab GTPases may be reversible through the action of protein phosphatases, which may reverse the signaling cascade. The four locations of phosphorylation were recently distinguished. For example, the phosphorylation at switch II may interfere with Rab–GAP interaction, simultaneously increasing or decreasing the interaction with the effector protein. On the other hand, phosphorylation within the α3/β5 loop antagonizes the catalytic activity of another kinase, LRRK2.^302^

It is the future task to comprehend how small GTPases are linked to diabetes and related disorders. In addition to the application of existing small molecular tools, continuously developing technologies, such as (phospho)proteome- and genome-wide screening, could be used as a measure to identify the various partners of small GTPases, including their mutual dependencies.

## Supporting Information Available

List of crystal structures of small GTPases playing a role in diabetes, corresponding to Figure 1; list of crystal structures of the enzymes of mevalonate pathway playing a role in diabetes mellitus, corresponding to Figure 3; amino acid sequence alignment of human GTPases involved in diabetes and insulin resistance (PDF)

## Abbreviations

Akt: protein kinase B
Arp2/3: actin-related protein 2/3 complex
BP: bisphosphonate
CD: cluster of differentiation
CRP: C-reactive protein
DKD: diabetic kidney disease
DMAPP: dimethylallyl pyrophosphate
ER: endoplasmic reticulum
ERGIC: ER-Golgi intermediate compartment
ERK: extracellular-signal-regulated kinase
FOH: farnesol
FPP: farnesyl pyrophosphate
FPPS: farnesyl pyrophosphate synthase
FTase: farnesyltransferase
GPP: geranyl pyrophosphate
FTI: FTase inhibitor
GGOH: geranylgeraniol
GGPP (or GRG): geranylgeranyl pyrophosphate, geranylgeranyl diphosphate
GGPPS: geranylgeranyl pyrophosphate synthase
GGTase-I: geranylgeranyltransferase type I
GGTase-II: Rab geranylgeranyl transferase
GGTase-III: geranylgeranyltransferase III
GLUT: glucose transporter
GSIS: glucose-stimulated insulin secretion
GSV: GLUT4 storage vesicles
HbA1c: hemoglobin A1c
HMG-CoA: 3-hydroxymethyl-3-methylglutaryl coenzyme A
ICAM-1: intracellular adhesion molecule 1
IgG: immunoglobulin G
IL: interleukin
IR: insulin resistance
ISG: insulin secretory granule
IPP: isopentenyl diphosphate
IR: insulin, resistance
IRS: insulin receptor substrate
IRV: insulin-responsive vesicles
Kir2: inwardly rectifying potassium channel 2
LFA-1: lymphocyte function-associated antigen
LPS: lipopolysaccharides
MCP-1: monocyte chemoattractant protein-1
MMP-1: matrix metalloproteinase-1
NAFLD: nonalcoholic fatty liver disease
NF-κB: nuclear factor kappa-light-chain-enhancer of activated B cells
NOX: NADPH oxidase
p65: LNR3NOD-like receptor family pyrin domain containing 3 inflammasome
PAK1: P21-activated kinase 1
PDK: phosphoinositide-dependent kinase
PHA: phytohemagglutinin
PM: plasma membrane
RGGT: Rab geranylgeranyl transferase
ROS: reactive oxygen species
RRP: readily releasable pool
SNARE: soluble N-ethylmaleimide sensitive factor attachment receptor
STZ: streptozotocin
SUR1: sulfonylurea receptor-1, a regulatory subunit of ATP-sensitive potassium channel
TCA cycle: tricarboxylic acid cycle
T2D: type 2 diabetes
TGN: Trans-Golgi Network
TNF-α: tumor necrosis factor α
VCAM-1: vascular cell adhesion molecule 1
VGCC: voltage-gated calcium channel
VSMC: vascular smooth muscle cell
