# Supplementary material for: Targeting Small GTPases and Their Prenylation in Diabetes Mellitus
Source: J Med Chem. 2021 Jul 8;64(14):9677–710. doi: 10.1021/acs.jmedchem.1c00410 (PMC8389838; doi:10.1021/acs.jmedchem.1c00410)
Supplement: Supplementary file 1 — jm1c00410_si_001.pdf [file jm1c00410_si_001.pdf]

Supplementary Information

Targeting small GTPases and their prenylation in diabetes mellitus

Edyta Gendaszewska-Darmach[1], Malgorzata A. Garstka[2]*, Katarzyna M. Błażewska[3]**

[1]*Institute of Molecular and Industrial Biotechnology, Faculty of Biotechnology and Food Sciences, Lodz University of Technology, Stefanowskiego 4/10, 90-924 Lodz, Poland;* edyta.gendaszewska-darmach@p.lodz.pl

[2]*Core Research Laboratory, Department of Endocrinology, Department of Tumor and Immunology, Precision Medical Institute, Western China Science and Technology Innovation Port, School of Medicine, the Second Affiliated Hospital of Xi'an Jiaotong, DaMingGong, Jian Qiang Road, Wei Yang district, Xi'an 710016, China,* m.garstka@xjtu.edu.cn

[3]*Institute of Organic Chemistry, Faculty of Chemistry Lodz University of Technology, Zeromskiego St. 116, 90-924 Lodz, Poland;* katarzyna.blazewska@p.lodz.pl

*Co-corresponding Authors

Content

1. Supplementary Figure S2-S6
2. Supplementary Table 1 S7-S10
3. Supplementary Table 2 S11-S13

A

P loop — G1 (positions 70–79); Switch I — G2 (position 96)

```
      .........10 .........20 .........30 .........40 .........50 .........60 .........70 .........80 .........90 .........100
Rab1a ---------- ---------- ---------- ---------- ---------- --MSSMNPEY DYLFKLLLIG DSGVGKSCLL LRFADDTY-T ESYISTIGVD
Rab35 ---------- ---------- ---------- ---------- ---------- -----MARDY DHLFKLLIIG DSGVGKSSLL LRFADNTF-S GSYITTIGVD
Rab13 ---------- ---------- ---------- ---------- ---------- -----MAKAY DHLFKLLLIG DSGVGKTCLI IRFAEDNF-N NTYISTIGID
Rab3a ---------- ---------- ---------- ---------- -MASATDSRY GQKESSDQNF DYMFKILIIG NSSVGKTSFL FRYADDSF-T PAFVSTVGID
Rab3b ---------- ---------- ---------- ---------- -MASVTDGKT GVKDASDQNF DYMFKLLIIG NSSVGKTSFL FRYADDTF-T PAFVSTVGID
Rab26 MSRKKTPKSK GASTPAASTL PTANGARPAR SGTALSGPDA PPNGPLQPGR PSLGGGVDFY DVAFKVMLVG DSGVGKTCLL VRFKDGAFLA GTFISTVGID
Rab37 ---------- ---------- ---------M WLMSEAHGAE PVLLREAARP FTQTLRLCVP SGNSKVMLLG DTGVGKTCFL IQFKDGAFLS GTFIATVGID
Rab8a ---------- ---------- ---------- ---------- ---------- -----MAKTY DYLFKLLLIG DSGVGKTCVL FRFSEDAF-N STFISTIGID
Rab10 ---------- ---------- ---------- ---------- ---------- ----MAKKTY DLLFKLLLIG DSGVGKTCVL FRFSDDAF-N TTFISTIGID
Rab24 ---------- ---------- ---------- ---------- ---------- -----MS-GQ RVDVKVVMLG KEYVGKTSLV ERYVHDRFLV GPYQNTIGAA
Rab7b ---------- ---------- ---------- ---------- ---------- -----MNPRK KVDLKLIIVG AIGVGKTSLL HQYVHKTF-Y EEYQTTLGAS
Rab18 ---------- ---------- ---------- ---------- ---------- -----MDEDV LTTLKILIIG ESGVGKSSLL LRFTDDTF-D PELAATIGVD
Rab27a---------- ---------- ---------- ---------- ---------- ----MSDGDY DYLIKFLALG DSGVGKTSVL YQYTDGKF-N SKFITTVGID
Rab7a ---------- ---------- ---------- ---------- ---------- -----MTSRK KVLLKVIILG DSGVGKTSLM NQYVNKKF-S NQYKATIGAD
Rab2a ---------- ---------- ---------- ---------- ---------- -------MAY AYLFKYIIIG DTGVGKSCLL LQFTDKRF-Q PVHDLTIGVE
Rab4a ---------- ---------- ---------- ---------- ---------- MSQTAMSETY DFLFKFLVIG NAGTGKSCLL HQFIEKKF-K DDSNHTIGVE
Rab11a---------- ---------- ---------- ---------- ---------- MGTR--DDEY DYLFKVVLIG DSGVGKSNLL SRFTRNEF-N LESKSTIGVE
Rab11b---------- ---------- ---------- ---------- ---------- MGTR--DDEY DYLFKVVLIG DSGVGKSNLL SRFTRNEF-N LESKSTIGVE
Rab14 ---------- ---------- ---------- ---------- ---------- MATA--PYNY SYIFKYIIIG DMGVGKSCLL HQFTEKKF-M ADCPHTIGVE
Rab4b ---------- ---------- ---------- ---------- ---------- M-----AETY DFLFKFLVIG SAGTGKSCLL HQFIENKF-K QDSNHTIGVE
Rab28 ---------- ---------- ---------- ---------- ---------- MSDS-EEESQ DRQLKIVVLG DGASGKTSLT TCFAQETF-G KQYKQTIGLD
```

Switch II — G3 (positions 159–163)

```
      .........110.........120.........130.........140.........150.........160.........170.........180.........190.........200
Rab1a FKIRTIELD- ---------G K-TIKLQIW- ---------- ---------- --------DT AGQERFRTIT SSYYRGAHGI IVVYDVTDQE SFNNVKQWL-
Rab35 FKIRTVEIN- ---------G E-KVKLQIW- ---------- ---------- --------DT AGQERFRTIT STYYRGTHGV IVVYDVTSAE SFVNVKRWL-
Rab13 FKIRTVDIE- ---------G K-KIKLQVW- ---------- ---------- --------DT AGQERFKTIT TAYYRGAMGI ILVYDITDEK SFENIQNWM-
Rab3a FKVKTIYRN- ---------D K-RIKLQIW- ---------- ---------- --------DT AGQERYRTIT TAYYRGAMGF ILMYDITNEE SFNAVQDWS-
Rab3b FKVKTVYRH- ---------E K-RVKLQIW- ---------- ---------- --------DT AGQERYRTIT TAYYRGAMGF ILMYDITNEE SFNAVQDWA-
Rab26 FRNKVLDVD- ---------G V-KVKLQMW- ---------- ---------- --------DT AGQERFRSVT HAYYRDAHAL LLLYDVTNKA SFDNIQAWL-
Rab37 FRNKVVTVD- ---------G V-RVKLQIW- ---------- ---------- --------DT AGQERFRSVT HAYYRDAQAL LLLYDITNKS SFDNIRAWL-
Rab8a FKIRTIELD- ---------G K-RIKLQIW- ---------- ---------- --------DT AGQERFRTIT TAYYRGAMGI MLVYDITNEK SFDNIRNWI-
Rab10 FKIKTVELQ- ---------G K-KIKLQIW- ---------- ---------- --------DT AGQERFHTIT TSYYRGAMGI MLVYDITNGK SFENISKWL-
Rab24 FVAKVMSVG- ---------D R-TVTLGIW- ---------- ---------- --------DT AGSERYEAMS RIYYRGAKAA IVCYDLTDSS SFERAKFWV-
Rab7b ILSKIIILG- ---------D T-TLKLQIW- ---------- ---------- --------DT GGQERFRSMV STFYKGSDGC ILAFDVTDLE SFEALDIWR-
Rab18 FKVKTISVD- ---------G N-KAKLAIWV TLHQQTANFF LKSQIGNSPI LKWAMWQYDT AGQERFRTLT PSYYRGAQGV ILVYDVTRRD TFVKLDNWL-
Rab27aFREKRVVYRA SGPDGATGRG Q-RIHLQLW- ---------- ---------- --------DT AGQERFRSLT TAFFRDAMGF LLLFDLTNEQ SFLNVRNWI-
Rab7a FLTKEVMVD- ---------D R-LVTMQIW- ---------- ---------- --------DT AGQERFQSLG VAFYRGADCC VLVFDVTAPN TFKTLDSWR-
Rab2a FGARMITID- ---------G K-QIKLQIW- ---------- ---------- --------DT AGQESFRSIT RSYYRGAAGA LLVYDITRRD TFNHLTTWL-
Rab4a FGSKIINVG- ---------G K-YVKLQIW- ---------- ---------- --------DT AGQERFRSVT RSYYRGAAGA LLVYDITSRE TYNALTNWL-
Rab11aFATRSIQVD- ---------G K-TIKAQIW- ---------- ---------- --------DT AGQERYRAIT SAYYRGAVGA LLVYDIAKHL TYENVERWL-
Rab11bFATRSIQVD- ---------G K-TIKAQIW- ---------- ---------- --------DT AGQERYRAIT SAYYRGAVGA LLVYDIAKHL TYENVERWL-
Rab14 FGTRIIEVS- ---------G Q-KIKLQIW- ---------- ---------- --------DT AGQERFRAVT RSYYRGAAGA LMVYDITRRS TYNHLSSWL-
Rab4b FGSRVVNVG- ---------G K-TVKLQIW- ---------- ---------- --------DT AGQERFRSVT RSYYRGAAGA LLVYDITSRE TYNSLAAWL-
Rab28 FFLRRITLP- ---------G NLNVTLQIW- ---------- ---------- --------DI GGQTIGGKML DKYIYGAQGV LLVYDITNYQ SFENLEDWYT
```

```
                       G4                                        G5
       .........210.........220.........230.........240.........250..  ....260.........270.........280.........290.........300
Rab1a  --QEIDRYAS ---EN-VNKL LVGNKCDLT- ---TKKVVDY TTAKEFADSL GIPFLETSAK NATNVEQSFM TMAAEIKKRM GPGA------ --TAGGAEKS
Rab35  --HEINQNC- ---DD-VCRI LVGNKNDDP- ---ERKVVET EDAYKFAGQM GIQLFETSAK ENVNVEEMFN CITELVLRAK KDNL------ --AKQQQQQQ
Rab13  --KSIKENAS ---AG-VERL LLGNKCDME- ---AKRKVQK EQADKLAREH GIRFFETSAK SSMNVDEAFS SLARDILLKS GGRR------ --SGNGNKPP
Rab3a  --TQIKTYSW ---DN-AQVL LVGNKCDME- ---DERVVSS ERGRQLADHL GFEFFEASAK DNINVKQTFE RLVDVICEKM SESL----DT ADPAVTGAKQ
Rab3b  --TQIKTYSW ---DN-AQVI LVGNKCDME- ---EERVVPT EKGQLLAEQL GFDFFEASAK ENISVRQAFE RLVDAICDKM SDSL-----D TDPSMLGSSK
Rab26  --TEIHEYAQ ---HD-VALM LLGNKVDSA- ---HERVVKR EDGEKLAKEY GLPFMETSAK TGLNVDLAFT AIAKELKQRS MKAP------ ------SEPR
Rab37  --TEIHEYAQ ---RD-VVIM LLGNKADMS- ---SERVIRS EDGETLAREY GVPFLETSAK TGMNVELAFL AIAKELKYRA GHQA------ ------DEPS
Rab8a  --RNIEEHAS ---AD-VEKM ILGNKCDVN- ---DKRQVSK ERGEKLALDY GIKFMETSAK ANINVENAFF TLARDIKAKM DKKL------ EGNSPQGSNQ
Rab10  --RNIDEHAN ---ED-VERM LLGNKCDMD- ---DKRVVPK GKGEQIAREH GIRFFETSAK ANINIEKAFL TLAEDILRKT PVKE------ ----PNSENV
Rab24  --KEL-RSLE ---EG-CQIY LCGTKSDLLE EDRRRRRVDF HDVQDYADNI KAQLFETSSK TGQSVDELFQ KVAEDYVSVA AFQV------ -----MTEDK
Rab7b  --GDVLAKIV PMEQS-YPMV LLGNKIDLA- ----DRKVPQ EVAQGWCREK DIPYFEVSAK NDINVVQAFE MLASRALSRY QSIL------ ------ENHL
Rab18  --NELETYCT ---RNDIVNM LVGNKIDKE- ----NREVDR NEGLKFARKH SMLFIEASAK TCDGVQCAFE ELVEKIIQTP GLWE------ ----SENQNK
Rab27a--SQLQMHAY ---CENPDIV LCGNKSDLE- ---DQRVVKE EEAIALAEKY GIPYFETSAA NGTNISQAIE MLLDLIMKRM ERCV----DK SWIPEGVVRS
Rab7a  --DEFLIQAS PRDPENFPFV VLGNKIDLE- ---NRQVATK RAQAWCYSKN NIPYFETSAK EAINVEQAFQ TIARNALKQE TEVE------ ---LYNEFPE
Rab2a  --EDARQHS- ---NSNMVIM LIGNKSDLE- ---SRREVKK EEGEAFAREH GLIFMETSAK TASNVEEAFI NTAKEIYEKI QEGVFDINNE ANGIKIGPQH
Rab4a  --TDARMLA- ---SQNIVII LCGNKKDLD- ---ADREVTF LEASRFAQEN ELMFLETSAL TGENVEEAFV QCARKILNKI ESGELD-PER MGSGIQYGDA
Rab11a--KELRDHA- ---DSNIVIM LVGNKSDLR- ---HLRAVPT DEARAFAEKN GLSFIETSAL DSTNVEAAFQ TILTEIYRIV SQKQM--SDR RENDMSPSNN
Rab11b--KELRDHA- ---DSNIVIM LVGNKSDLR- ---HLRAVPT DEARAFAEKN NLSFIETSAL DSTNVEEAFK NILTEIYRIV SQKQI--ADR AAHDESPGNN
Rab14  --TDARNLT- ---NPNTVII LIGNKADLE- ---AQRDVTY EEAKQFAEEN GLLFLEASAK TGENVEDAFL EAAKKIYQNI QDGSL--DLN AAESGVQHKP
Rab4b  --TDARTLA- ---SPNIVVI LCGNKKDLD- ---PEREVTF LEASRFAQEN ELMFLETSAL TGENVEEAFL KCARTILNKI DSGEL--DPE RMGSGIQYGD
Rab28  VVKKVSEES- ---ETQPLVA LVGNKIDLE- ---HMRTIKP EKHLRFCQEN GFSSHFVSAK TGDSVFLCFQ KVAAEILGIK LNKAE----I EQSQRVVKAD
```

```
      .........310.........320.....
Rab1a NVKIQSTPVK Q-SG---GGC -C---
Rab35 NDVVKLTKNS K-RK---KRC -C---
Rab13 STDLKTCDK- K-NT---NKC -SLG-
Rab3a GPQLSDQQVP P-HQ---DCA -C---
Rab3b NTRLSDTPPL L-QQ---NCS -C---
Rab26 FRLHDYVKRE G-RG---ASC -CRP-
Rab37 FQIRDYVESQ K-KR---SSC -CSFM
Rab8a GVKITPDQQK R-SS---FFR -CVLL
Rab10 DISSGGGVT- G-WK---SKC -C---
Rab24 GVDLGQKPN- P-YF---YSC -CHH-
Rab7b TESIKLSPD- Q-SR---SRC -C---
Rab18 GVKLSHREEG Q-GGGACGGY -CSVL
Rab27aNGHASTDQLS E-EKE--KGA -CGC-
Rab7a PIKLDKNDRA K-AS---AES -CSC-
Rab2a AATNATHAGN QGGQQAGGGC -C---
Rab4a ALRQLRSPRR AQAPNAQECG -C---
Rab11aVVPIHVPPTT E--NKPKVQC -CQNI
Rab11bVVDISVPPTT DGQKPNKLQC -CQNL
Rab14 SAPQGGRLTS EPQPQREGCG -C---
Rab4b ASLRQLRQPR SAQAVAPQPC GC---
Rab28 IVNYNQEPMS RTVNPPRSSM -CAVQ
```

Prenylation

1

B

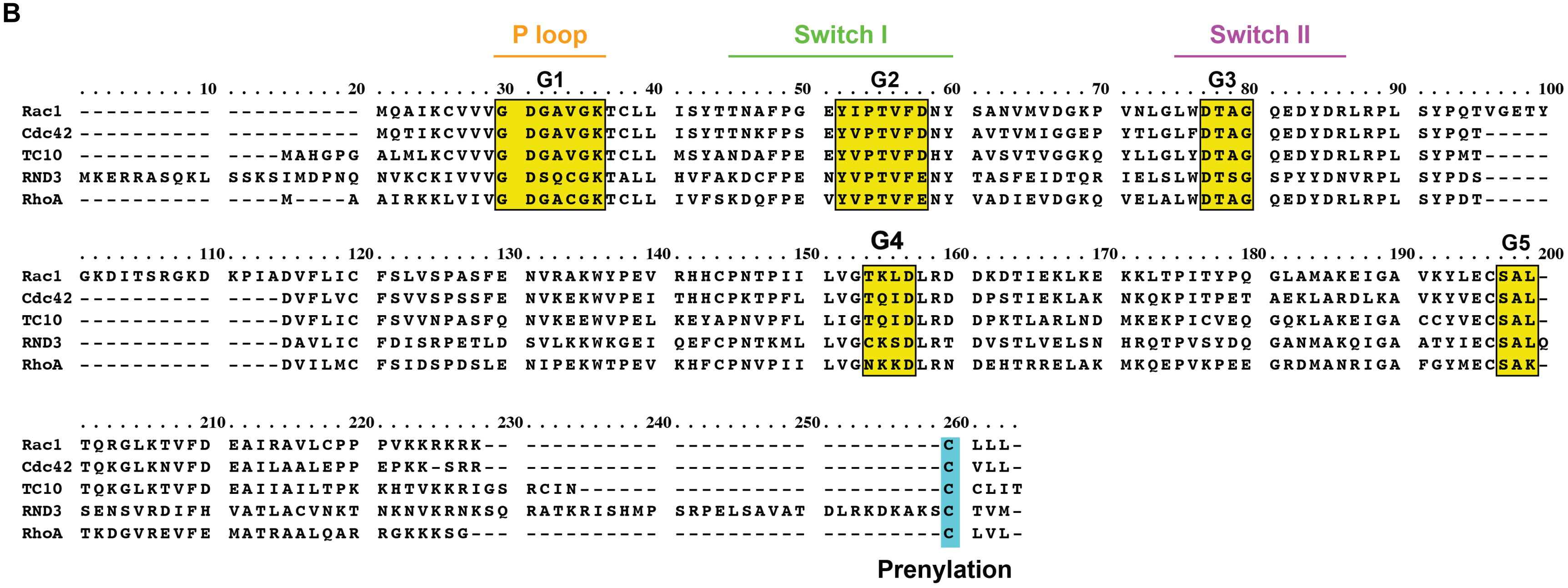
P loop
Switch I
Switch II
G1
G2
G3
10 20 30 40 50 60 70 80 90 100
Rac1 ---------- ---------- MQAIKCVVVG DGAVGKTCLL ISYTTNAFPG EYIPTVFDNY SANVMVDGKP VNLGLWDTAG QEDYDRLRPL SYPQTVGETY
Cdc42 ---------- ---------- MQTIKCVVVG DGAVGKTCLL ISYTTNKFPS EYVPTVFDNY AVTVMIGGEP YTLGLFDTAG QEDYDRLRPL SYPQT-----
TC10 ---------- ----MAHGPG ALMLKCVVVG DGAVGKTCLL MSYANDAFPE EYVPTVFDHY AVSVTVGGKQ YLLGLYDTAG QEDYDRLRPL SYPMT-----
RND3 MKERRASQKL SSKSIMDPNQ NVKCKIVVVG DSQCGKTALL HVFAKDCFPE NYVPTVFENY TASFEIDTQR IELSLWDTSG SPYYDNVRPL SYPDS-----
RhoA ---------- ----M----A AIRKKLVIVG DGACGKTCLL IVFSKDQFPE VYVPTVFENY VADIEVDGKQ VELALWDTAG QEDYDRLRPL SYPDT-----
G4
G5
110 120 130 140 150 160 170 180 190 200
Rac1 GKDITSRGKD KPIADVFLIC FSLVSPASFE NVRAKWYPEV RHHCPNTPII LVGTKLDLRD DKDTIEKLKE KKLTPITYPQ GLAMAKEIGA VKYLECSAL-
Cdc42 ---------- ----DVFLVC FSVVSPSSFE NVKEKWVPEI THHCPKTPFL LVGTQIDLRD DPSTIEKLAK NKQKPITPET AEKLARDLKA VKYVECSAL-
TC10 ---------- ----DVFLIC FSVVNPASFQ NVKEEWVPEL KEYAPNVPFL LIGTQIDLRD DPKTLARLND MKEKPICVEQ GQKLAKEIGA CCYVECSAL-
RND3 ---------- ----DAVLIC FDISRPETLD SVLKKWKGEI QEFCPNTKML LVGCKSDLRT DVSTLVELSN HRQTPVSYDQ GANMAKQIGA ATYIECSALQ
RhoA ---------- ----DVILMC FSIDSPDSLE NIPEKWTPEV KHFCPNVPII LVGNKKDLRN DEHTRRELAK MKQEPVKPEE GRDMANRIGA FGYMECSAK-
210 220 230 240 250 260
Rac1 TQRGLKTVFD EAIRAVLCPP PVKKRKRK-- ---------- ---------- ---------C LLL-
Cdc42 TQKGLKNVFD EAILAALEPP EPKK-SRR-- ---------- ---------- ---------C VLL-
TC10 TQKGLKTVFD EAIIAILTPK KHTVKKRIGS RCIN------ ---------- ---------C CLIT
RND3 SENSVRDIFH VATLACVNKT NKNVKRNKSQ RATKRISHMP SRPELSAVAT DLRKDKAKSC TVM-
RhoA TKDGVREVFE MATRAALQAR RGKKKSG--- ---------- ---------- ---------C LVL-
Prenylation

C

P loop G1

```
      .........10 .........20 .........30 .........40 .........50 .........60 .........70 .........80 .........90 .........100
RalA  ---------- ---------- ---------- ---------- ---------- ---------- ---------- -------MAA NKPKGQNSLA LHKVIMVGSG
RRAD  MTLNGGGSGA GGSRGGGQER ERRRGSTPWG PAPPLHRRSM PVDERDLQAA LTPGALTAAA AGTGTQGPRL DWPEDSEDSL SSGGSDSDES VYKVLLLGAP
HRas  ---------- ---------- ---------- ---------- ---------- ---------- ---------- ---------- --------MT EYKLVVVGAG
NRas  ---------- ---------- ---------- ---------- ---------- ---------- ---------- ---------- --------MT EYKLVVVGAG
KRAs  ---------- ---------- ---------- ---------- ---------- ---------- ---------- ---------- --------MT EYKLVVVGAG
Rap1a ---------- ---------- ---------- ---------- ---------- ---------- ---------- ---------- --------MR EYKLVVLGSG
Rap1b ---------- ---------- ---------- ---------- ---------- ---------- ---------- ---------- --------MR EYKLVVLGSG
```

P loop G1 — Switch I G2 — Switch II G3

```
      .........110.........120.........130.........140.........150.........160.........170.........180.........190.........200
RalA  GVGKSALTLQ FMYDEFVEDY EPTKADSYRK KVVLDGEEVQ IDILDTAGQE DYAAIRDNYF RSGEGFLCVF SITEMESFAA TADFREQILR VKEDENVPFL
RRAD  GVGKSALARI FGGVED-GPE AEAAGHTYDR SIVVDGEEAS LMVYDIWEQD GGRWLPGHCM AMGDAYVIVY SVTDKGSFEK ASELRVQLRR ARQTDDVPII
HRas  GVGKSALTIQ LIQNHFVDEY DPTIEDSYRK QVVIDGETCL LDILDTAGQE EYSAMRDQYM RTGEGFLCVF AINNTKSFED IHQYREQIKR VKDSDDVPMV
NRas  GVGKSALTIQ LIQNHFVDEY DPTIEDSYRK QVVIDGETCL LDILDTAGQE EYSAMRDQYM RTGEGFLCVF AINNSKSFAD INLYREQIKR VKDSDDVPMV
KRAs  GVGKSALTIQ LIQNHFVDEY DPTIEDSYRK QVVIDGETCL LDILDTAGQE EYSAMRDQYM RTGEGFLCVF AINNTKSFED IHHYREQIKR VKDSEDVPMV
Rap1a GVGKSALTVQ FVQGIFVEKY DPTIEDSYRK QVEVDCQQCM LEILDTAGTE QFTAMRDLYM KNGQGFALVY SITAQSTFND LQDLREQILR VKDTEDVPMI
Rap1b GVGKSALTVQ FVQGIFVEKY DPTIEDSYRK QVEVDAQQCM LEILDTAGTE QFTAMRDLYM KNGQGFALVY SITAQSTFND LQDLREQILR VKDTDDVPMI
```

G4 — G5

```
      .........210.........220.........230.........240.........250.........260.........270.........280.........290.........300
RalA  LVGNKSDLED KRQVSVEEAK NRAEQWN-VN YVETSAKTRA NVDKVFFDLM REIRARKMED SKEKNGKKKR ---------- ---------- --KSLAKRIR
RRAD  LVGNKSDLVR SREVSVDEGR ACAVVFD-CK FIETSAALHH NVQALFEGVV RQIRLRRDSK EANARRQAGT RRRESLGKKA KRFLGRIVAR NSRKMAFRAK
HRas  LVGNKCDLAA -RTVESRQAQ DLARSYG-IP YIETSAKTRQ GVEDAFYTLV REIRQHKLRK LNPPD----- ---------- ---------- --ESGPGCMS
NRas  LVGNKCDLPT -RTVDTKQAH ELAKSYG-IP FIETSAKTRQ GVEDAFYTLV REIRQYRMKK LNSSD----- ---------- ---------- --DGTQGCMG
KRAs  LVGNKCDLPS -RTVDTKQAQ DLARSYG-IP FIETSAKTRQ RVEDAFYTLV REIRQYRLKK ISKEE----- ---------- ---------- --KTPGCVKI
Rap1a LVGNKCDLED ERVVGKEQGQ NLARQWCNCA FLESSAKSKI NVNEIFYDLV RQINRKTPVE KKKPK----- ---------- ---------- --KK------
Rap1b LVGNKCDLED ERVVGKEQGQ NLARQWNNCA FLESSAKSKI NVNEIFYDLV RQINRKTPVP GKARK----- ---------- ---------- --KS------
```

```
      .........310
RalA  ERCC----IL
RRAD  SKSCHDLSVL
HRas  CKCV----LS
NRas  LPCV----VM
KRAs  KKCI----IM
Rap1a -SCL----LL
Rap1b -SCQ----LL
```

Prenylation

Supplementary Figure 1 corresponding to Figure 1. Amino acid sequence alignment of human GTPases involved in diabetes and insulin resistance

Protein sequences were downloaded from String: functional proteins association network. Alignment of Rab (A), Rho (B) and Ras (C) proteins was performed using Praline (developed in the Centre for Integrative Bioinformatics Vrije Universteit Amsterdam). Important regions are indicated: the P loop (orange), switch I (green) and switch II (magenta), the nucleotide-binding G-motifs (yellow, G1-G5) and the C-terminal cysteine important for prenylation (cyan). Numbers at the top indicate the residue number.

Supplementary Table 1 corresponding to Figure 1. List of crystal structures of small GTPases playing a role in diabetes.

GTPase	GDP-bound	GTP[&]-bound	In complex with effector	In complex with GEF	In complex with GAD
**Rab GTPases**					
Rab1a	2FOL	ND	ND^	ND^	ND^
Rab2a	ND	ND	ND	ND	ND
Rab3a	ND	3RAB	ND	ND	ND
Rab3b	3DZ8	ND	ND	ND	ND
Rab4a	2BMD	1YU9<br>2BME	ND	ND	ND
Rab4b	2O52	ND	ND	ND	ND
Rab5a	1TU4<br>2BMD	1N6H	ND^	ND^	ND
Rab7	**1VG1**	1T91<br>**1VG8**	**1YHN (RILP)**	ND	ND
Rab8a	4LHV<br>6STF	4LHW<br>6STG	ND^	ND^	ND^
Rab10	ND	ND	ND^	ND	ND
Rab11a	1OIV<br>1OIX (and Pi)<br>6IY1	1OIW<br>1YZK	ND^	ND^	ND

Rab11b	2F9L	2F9M	ND^	ND	ND
Rab13	ND	ND	ND	ND	ND
Rab14	1Z0F<br>4DRZ	ND	ND^	ND	ND
Rab18	ND	1X3S	ND	ND	ND
Rab24	ND	ND	ND	ND	ND
Rab26	ND	2G6B	ND	ND	ND
Rab27a	ND	HUF6	ND^	ND	ND
Rab28	2HXS	3E5H	ND	ND	ND
Rab35	ND#	ND	ND^	ND^	ND
Rab37	ND	ND	ND	ND	ND
**Rho GTPases**					
RhoA	1FTN<br>5E26<br>6KX2	1A2B<br>3TVD	ND^	ND^	ND
Cdc42	ND#	2QR2	1CEE (WASP)	ND^	ND^
Rac1	5N6O<br>**6AGP**	1MH1<br>**3TH5**	ND^	1FOE (TIAM1)<br>**4YON (P-Rex1)**<br>5O33 (Kalirin DH1)	ND^

TC10	ND	2ATX	2KE4 (CDc42-interacting domain of CIP4)	ND	ND
RND3/ RhoE	ND	1GWN (only core domain)<br>1M7B	ND^	ND	ND
			**Ras GTPases**		
RalA	1U90<br>1U8Z<br>6P0O<br>6P0J	ND	1UAD (Sec5)<br>1ZC3 (Exo84)<br>1ZC4 (Exo84)	ND	ND
(R)Rad	2DPX<br>2GJS	3Q72 (G domain)<br>3Q7P (G domain)	ND	ND	ND
Rap1	ND	ND	ND^	ND	ND
H-Ras	1CRR<br>1CRQ<br>1CRP<br>**2CLD**<br>4Q21	121P<br>1CTQ<br>1PLK<br>**1QRA**<br>3TGP<br>4RSG<br>4EFL<br>5P21	ND^	1NVX (SOS)<br>1NVW (SOS)<br>1NVU (SOS)<br>1BKD(SOS-1)<br>4URY(SOS)	**1WQ1 (RasGAP)**
N-Ras	ND	5UHV	ND	ND	ND

K-Ras	4OBE<br>5VQ8<br>5W22<br>6MBT<br>6MBU	5VQ2<br>5VQ6<br>6GOD<br>6VC8	ND^	ND	ND

Structures identification codes (PDB ID codes) were assessed from the Protein Data Bank, http://www.pdb.org. As source organism taxonomy was selected *Homo sapiens*, *Mus musculus*, and *Rattus norvegicus*. X-ray and NMR structures are included. NMR structures are underlined. Only the structures of GTPases with a single Mg^{2+} were included. Structures in the complex with the effectors unrelated to diabetes were omitted. ND - not determined for GTPases; ND^ - there exist crystal structures of GTPase-effector/GEF/GAP complexes, but unrelated to diabetes, insulin resistance or insulin-dependent tissue; ND[#] - crystal structure of GDP-bound form exists only in the in complex with GEF; [&]GTP or its non-hydrolyzable analog, including GppNHp, GTPgammaS, GMP-PCP; PDB ID codes in bold represent structures used for the generation of Figure 1.

Supplementary Table 2 corresponding to Figure 3. List of crystal structures of the enzymes of mevalonate pathway playing a role in diabetes mellitus

Enzyme	Only enzyme	With substrate/product	With inhibitor
HMG-CoA reductase	ND^	1DQ8 (HMG and CoA)<br>**1DQ9 (HMG-CoA)**<br>1DQA (HMG, CoA and NADP+)	1HW9 (Simvastatin)<br>1HWI (Fluvastatin)<br>1HWK (Artovastatin)
Farnesyl pyrophosphate synthase (FPPS)/ farnesyl diphosphate synthase (FDPS)	1ZW5<br>2F7M<br>4XQR<br>4XQS (Mg^{2+})<br>4XQT ($3Mg^{2+}$)	**5JA0 (FPP)**	2F89 (pamidronate)<br>2F8C (zoledronate)<br>2F92 (alendronate)
Geranylgeranyl diphosphate synthase (GGPPS)	ND^	**2Q80 (GGPP)**	ND^
Farnesyltransferase (FTase)	1FT1	**1FPP (FPP)**<br>1FT2 (FPP, Zn^{2+})<br>1JC6 (FPP, TKCVFM peptide substrate)<br>1KZP (farnesylated K-Ras4b)<br>1TN8 (FPP, H-Ras)<br>2H6F (farnesylated DDPTASACVLS peptide)	1SA4 (R115777)<br>1SA5 (BMS-214662)

Geranylgeranyltransferase-I (GGTase-I)	1DCE	**1N4P (GRG)**<br>1N4Q (GGPP*, geranylgeranylated KKKSKTKCVIL peptide)<br>1N4R (geranylgeranylated KKKSKTKCVIL peptide)<br>1N4S (GGPP, geranylgeranylated KKKSKTKCVIL peptide)<br>1TNO (GGPP*, geranylgeranylated KKKSKTKCVIM peptide from K-Ras4b)<br>1TNZ (GGPP*, geranylgeranylated RRCVLL peptide from Cdc42)	ND^
Geranylgeranyltransferase type-2 (GGTase-2)	3DSS	**3DST (GRG)**<br>3DSU (FPP, $Ca^{2+}Zn^{2+}$)<br>3DSV (monoprenylated SCSC peptide from Rab7)<br>3DSW (monoprenylated SCSC peptide from Rab7)<br>3DSX (diprenylated SCSC peptide from Rab7)	ND^
Geranylgeranyltransferase type-III (GGTase-III)	**6J6X**[&]	ND^	ND^

Supplementary Table 2 corresponding to Figure 3. List of crystal structures of the enzymes of mevalonate pathway playing a role in diabetes mellitus.

Structures identification codes (PDB ID codes) were assessed from the Protein Data Bank, http://www.pdb.org. As source organism taxonomy was selected *Homo sapiens*, *Mus musculus*, and *Rattus norvegicus*. Structures in the complex with the inhibitors unrelated to diabetes were omitted. ND - not determined for the enzymes, ND^ - there exist crystal structures of enzyme/substrate/product or

enzyme-inhibitor complexes, but unrelated to diabetes, insulin resistance or insulin-dependent tissue, *analog, [&] deposited, but unpublished. PDB ID codes in bold represent structures used for the generation of Figure 3.
